# The global, regional, and national burden of pediatric stone disease: 1990–2021 and projections for the next two decades

**DOI:** 10.3389/fped.2025.1529407

**Published:** 2025-03-25

**Authors:** Sheng Chen, Xiaohan Ma, Lin Guo, Shuaikang Wang, Junchao Wu, Lingling Wu, Ting Zhang, Hongjun Gao

**Affiliations:** ^1^ Graduate School, Guangxi University of Chinese Medicine, Nanning, Guangxi, China; ^2^ Ruikang Hospital, Guangxi University of Chinese Medicine, Nanning, Guangxi, China

**Keywords:** pediatric stone disease, GBD, incidence, mortality, DALYs, temporal trends

## Abstract

**Background:**

Pediatric stone disease, once considered rare, has gained significant attention over the past decade owing to its rapidly increasing incidence. Despite this surge, a comprehensive evaluation of this burden is lacking.

**Objectives:**

This study aimed to estimate the burden of pediatric stone disease, stratified by age and sex, at the global, regional, and national levels from 1990 to 2021.

**Methods:**

Data on the global incidence, deaths, and disability-adjusted life years (DALYs) related to pediatric stone disease from 1990 to 2021 were collected. The estimated annual percentage change (EAPC) quantified the disease trends over this period. Additionally, the relationship between disease burden and factors such as age and sociodemographic index (SDI) levels was analyzed. A Bayesian Age-Period-Cohort (BAPC) model was employed to project the future burden from 2022 to 2041.

**Results:**

In 2021, there were 3,289,663 cases of pediatric stone disease worldwide (95% UI: 1,724,296 to 5,384,797), resulting in 66 deaths (95% UI: 43 to 94) and 14,230 disabilities (95% UI: 9,264 to 21,569). Regionally, South Asia reported the highest incidence, mortality, and DALYs based on the Global Burden of Disease (GBD) classifications. Age-standardized morbidity (ASIR) and age-standardized mortality (ASDR) are highest in Eastern Europe, while age-standardized mortality (ASMR) is 0 in all regions of the world. At the country level, India recorded the highest incidence, mortality, and DALYs for pediatric stone disease in 2021. Armenia had the highest ASIR, while 28 countries, including Afghanistan, Armenia, and Brazil, reported the highest ASMR. Armenia and Kazakhstan recorded the highest ASDR. The disease burden was most pronounced among children aged 15–19 years, with boys being more affected than girls. These findings have significant global implications. Projections indicate that by 2041, the burden of pediatric stone disease will decline, although boys will continue to be more affected than girls.

**Conclusion:**

From 1990 to 2021, the global burden of pediatric stone disease, adjusted for age, has decreased. However, regional variations persist, with some areas experiencing an increase in burden. This underscores the importance of ongoing monitoring to effectively reduce the overall impact of pediatric stone diseases.

## 1 Introduction

Urolithiasis, defined by the formation or presence of mineral deposits in the urinary tract, excluding nephrocalcinosis, can be classified as nephrolithiasis, ureterolithiasis, or cystolithiasis. In children, this condition is referred to as pediatric stone disease (1). The global incidence of pediatric stone disease is increasing, driven not only by lifestyle and dietary changes but also by the increased use of imaging modalities (2, 3). The etiology of the disease is multifactorial, involving environmental, metabolic, anatomical, infectious, nutritional, and genetic factors (4–7). Approximately 75% of urinary stones form in the kidneys (5, 8, 9). Bladder stones, although less common, can originate within the bladder or descend from the upper urinary tract. Their occurrence is often linked to malnutrition (particularly protein-poor diets), early carbohydrate feeding, insufficient milk supply in newborns, the presence of foreign bodies, or bladder surgery (10). In our study, only 0.3% of the patients had bladder stones. The number of stones analyzed in children remains low, primarily because stones are often passed at home, and families may not recognize the importance of stone analysis (8, 11). Calcium is the most frequently identified mineral in stone analyses, with calcium oxalate stones being the most common (2, 8, 9, 11–14). Pediatric stone disease presents distinctively from its adult counterpart, requiring individualized medical and surgical treatments to minimize recurrence and safeguard renal function (15). While renal colic and macroscopic hematuria—common in adult cases—are rare in children, pediatric patients often present with few or no symptoms (7, 12, 16).

Approximately 50%–75% of urinary stones in children result from metabolic abnormalities, leading to high rates of recurrence and kidney damage (4–7, 17). However, disparities in disease burden among pediatric patients with stone disease across countries, regions, and ethnic groups remain poorly understood. This highlights the need to prioritize prevention, diagnosis, and treatment strategies for this population. Using the GBD statistical model, we assessed the global impact of pediatric stone disease in 2021 by examining the prevalence, incidence rates, mortality figures, and DALYs by age, sex, and temporal trends. We also projected the morbidity and mortality for the next two decades. These findings offer valuable insights for clinicians, epidemiologists, and health policymakers, supporting the optimization of resource allocation and the development of effective public health strategies.

## 2 Methods

### 2.1 Data source

This study conducted a secondary analysis using publicly available anonymized aggregate data from the GBD 2021. The dataset evaluated the burden of pediatric stone disease across 204 countries and territories from 1990 to 2021. GBD 2021 offers a standardized framework for integrating, validating, analyzing, and disseminating data on disease burdens while assessing the impact of premature death, health loss, and disability across diverse populations. The GBD generates estimates such as incidence, prevalence, mortality, and DALYs across all regions using a wide range of data sources, including administrative hospital and medical claims records, cause-of-death data, and both published and unpublished literature (18).

We applied the Das Gupta decomposition method to analyze the changes in the burden of Pediatric Stone Disease from 1990 to 2021. This method allowed us to attribute the overall changes in disease burden to factors such as population aging, population growth, and shifts in disease prevalence. By doing so, we gained a clearer understanding of how demographic and epidemiological changes have influenced the trends over the past three decades (19).

In this study, we collected and analyzed data on the global incidence, mortality, and DALYs associated with pediatric stone disease from 1990 to 2021, stratified by sex. DALYs, a summary measure of disease burden used in the GBD study, quantify the impact of both disability and premature death (20). They are calculated by summing the years of life lost (YLLs) due to premature death and years lived with disability (YLDs), with one DALY representing one lost year of a healthy life. Data for this analysis were obtained using the Global Health Data Exchange (GHDx) query tool (http://ghdx.healthdata.org/). To ensure transparency, reliability, and replicability, we followed the Guidelines for Accurate and Transparent Health Estimates Reporting (GATHER) (21). Additionally, we explored the relationship between the socio-demographic index (SDI) and disease burden. The SDI, a composite index reflecting a country's per capita income, average years of education, and fertility rate, categorizes 204 countries and regions into five groups: low SDI (<0.45), medium-low SDI (≥0.45 and <0.61), medium SDI (≥0.61 and <0.69), medium-high SDI (≥0.69 and <0.80), and high SDI (≥ 0.80) (22, 23).

### 2.2 Statistical analyses

We analyzed the incidence, mortality, and DALYs by examining the number, percentage, and rate of cases, with 95% uncertainty intervals (UIs) provided for each estimate (20). To assess temporal trends in the burden of pediatric stone disease from 1990 to 2021, we calculated EAPC in age-standardized rates (ASRs) for incidence, mortality, and DALYs. The EAPC is a widely accepted metric for quantifying changes in ASRs over time. This was calculated using a regression model fitted to the natural logarithm of the rate. The model was defined as ln(rate) = α + β × (calendar year) + ε, where EAPC was derived as 100 × [exp(β)—1]. The 95% confidence interval (CI) was calculated using this linear regression model. The 95% CI was calculated using this linear regression model. If both the EAPC and lower bound of the 95% CI are positive, the ASR is considered to increase. Conversely, if both the EAPC and the upper bound of the 95% CI are negative, the ASR is considered to decline. We also used the Pearson correlation coefficient (ρ) to examine the relationship between ASMR, ASDR, and SDI, assessing the impact of socioeconomic factors on the disease burden. The GBD 2021 analysis adjusted these Pearson correlation calculations to account for differences in population size and case frequency across countries and regions. Additionally, we employed the BAPC model to project the disease burden from 2022 to 2041 (24). All statistical analyses were performed using R software.

## 3 Results

### 3.1 Global burden of pediatric stone disease

**Figure 1:**
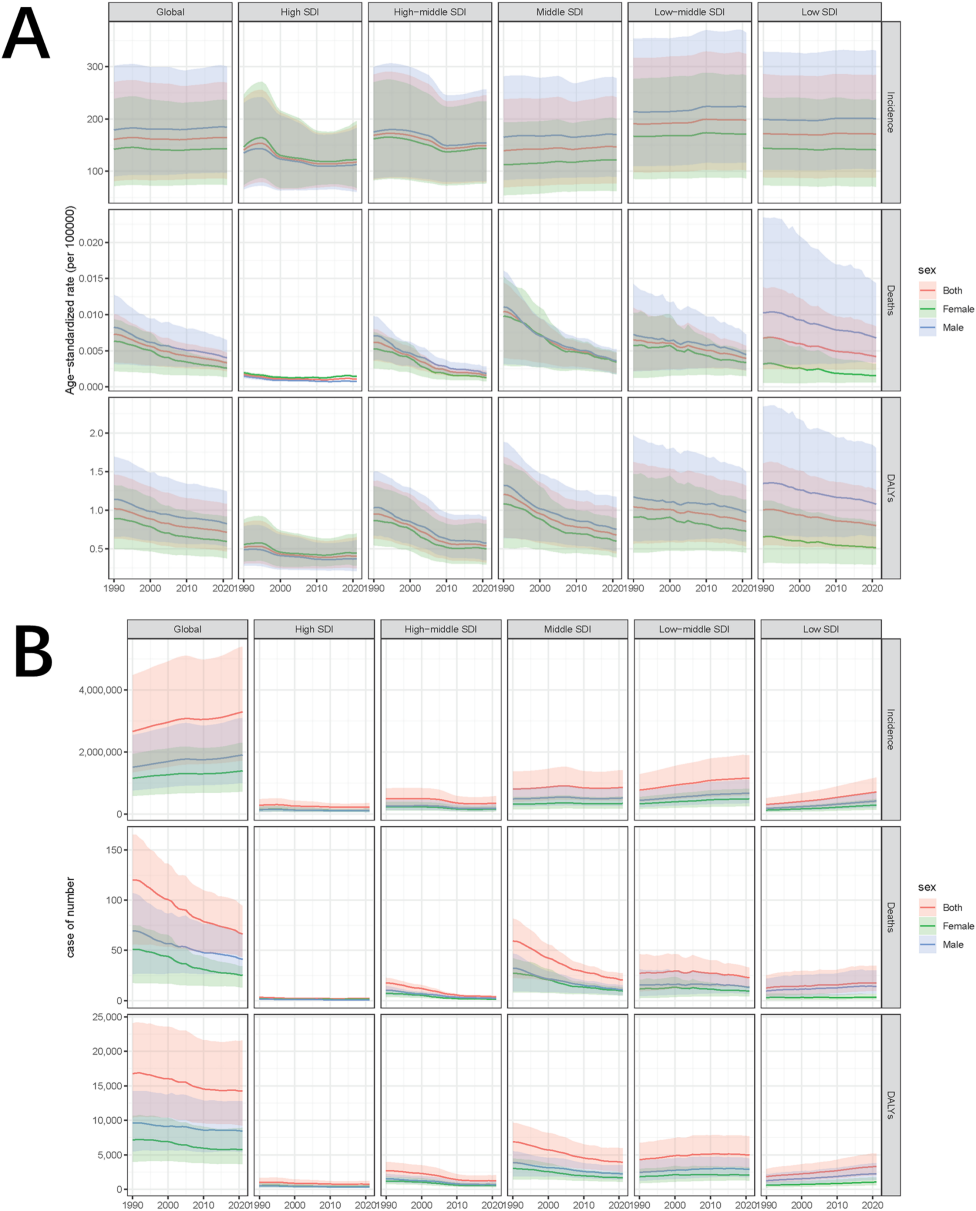
Temporal trends of ASIR, ASMR, and ASDR in pediatric stone disease, 1990–2021. **(A)** ASIR, ASMR, and ASDR of pediatric stone disease by sex, globally and in five SDI regions. **(B)** The number of cases of pediatric stone disease, disaggregated by sex, globally, and in the five SDI regions.

From 1990 to 2021, the global incidence of pediatric stone disease has increased, whereas mortality and DALYs have decreased (Figure 1A). In 2021, 3,289,663 cases of pediatric stone disease (95% UI: 1,724,296 to 5,384,797), 66 deaths (95% UI: 43 to 94), and 14,230 DALYs (95% UI: 9,264 to 21,569) were reported worldwide. The number of cases rose from 2,659,391 in 1990 to 3,289,663 in 2021, while deaths declined from 120 in 1990 to 66 in 2021, and DALYs dropped from 16,751 to 14,230 during the same period (Tables 1–3). The trend in ASR for pediatric stone disease mirrors that of incidence, mortality, and DALYs (Figure 1B). The ASIR increased from 160.87 (95% UI: 81.45 to 270.11) per 100,000 population in 1990 to 164.27 (95% UI: 86.05 to 269.03) per 100,000 in 2021. In contrast, the ASMR declined from 0.01 (95% UI: 0 to 0.01) per 100,000 population in 1990 to 0 (95% UI: 0 to 0) per 100,000 population in 2021. And the ASDR decreased from 1.02 (95% UI: 0.63 to 1.46) per 100,000 in 1990 to 0.71 (95% UI: 0.46 to 1.08) per 100,000 in 2021 (Tables 1–3).

**Table 1:** Incidence of pediatric stone disease in 1990 and 2021 for both sexes and all locations, with EAPC from 1990 to 2021.

location	Num_1990	ASR_1990	Num_2021	ASR_2021	EAPC_CI
Andean Latin America	18,209 (9,239 to 30,680)	134.5 (68.32 to 226.6)	25,114 (13,964 to 40,267)	141.02 (78.33 to 226.18)	0.31% (0.24 to 0.38)
Australasia	3,898 (1,713 to 6,946)	76.11 (33.15 to 135.61)	4,243 (1,942 to 7,476)	72.72 (33.21 to 128.16)	−0.16% (−0.22 to −0.1)
Caribbean	14,617 (7,203 to 24,990)	129.58 (63.69 to 221.48)	15,328 (7,585 to 26,423)	131.34 (64.83 to 226.46)	0.05% (0.04 to 0.06)
Central Asia	77,597 (41,383 to 128,632)	356.7 (190.16 to 591.23)	85,413 (46,031 to 139,620)	357.87 (192.87 to 584.89)	0.04% (0.01 to 0.07)
Central Europe	115,502 (62,348 to 189,012)	373.77 (201.9 to 612.13)	60,842 (35,330 to 93,771)	329.7 (191.66 to 508.73)	−0.59% (−0.7 to −0.49)
Central Latin America	83,647 (42,170 to 142,706)	140.29 (70.73 to 239.41)	89,307 (47,537 to 148,181)	134.15 (71.37 to 222.57)	−0.26% (−0.62 to 0.11)
Central Sub-Saharan Africa	26,259 (13,211 to 44,509)	133.05 (67.26 to 224.99)	69,160 (34,639 to 117,483)	135.73 (68.18 to 230.36)	0.08% (0.07 to 0.09)
East Asia	343,721 (159,159 to 616,754)	92.14 (42.17 to 165.23)	143,047 (68,455 to 244,601)	55.19 (26.47 to 94.35)	−2.16% (−2.48 to −1.83)
Eastern Europe	200,812 (107,480 to 328,462)	397.46 (212.68 to 650.03)	138,006 (73,570 to 225,260)	386.64 (206.25 to 631.31)	−0.13% (−0.14 to −0.11)
Eastern Sub-Saharan Africa	112,870 (57,624 to 187,451)	158.38 (81.23 to 262.56)	256,790 (131,167 to 425,684)	158.17 (80.87 to 262.16)	−0.04% (−0.06 to −0.01)
Global	26,59,391 (1,347,418 to 4,466,920)	160.87 (81.45 to 270.11)	32,89,663 (1,724,296 to 5,384,797)	164.27 (86.05 to 269.03)	0.01% (−0.02 to 0.05)
High-income Asia Pacific	63,665 (31,455 to 108,098)	146.69 (72.06 to 249.17)	30,503 (15,601 to 50,194)	119.91 (61.1 to 197.66)	−0.98% (−1.18 to −0.78)
High-income North America	95,487 (46,804 to 162,463)	151.87 (74.16 to 258.67)	96,489 (55,232 to 150,929)	130.24 (74.23 to 203.83)	−1.31% (−1.8 to −0.83)
High-middle SDI	490,251 (246,203 to 827,622)	168.82 (84.6 to 284.76)	349,812 (185,645 to 574,372)	149.15 (79.14 to 244.93)	−0.68% (−0.81 to −0.56)
High SDI	281,238 (140,103 to 475,275)	140.72 (69.86 to 237.8)	220,387 (120,101 to 354,389)	117.63 (63.93 to 189.15)	−1% (−1.19 to −0.81)
Low-middle SDI	771,718 (397,469 to 1,281,377)	190.72 (98.37 to 316.41)	11,57,622 (597,885 to 1,896,931)	197.65 (101.97 to 324.11)	0.18% (0.15 to 0.2)
Low SDI	307,195 (157,483 to 511,523)	171.91 (88.49 to 285.81)	705,547 (361,583 to 1,171,668)	170.99 (87.74 to 283.9)	0.02% (0 to 0.04)
Middle SDI	806,015 (401,096 to 1,373,431)	139.37 (69.24 to 237.39)	853,925 (446,831 to 1,402,177)	146.72 (76.71 to 241.04)	0.14% (0.12 to 0.17)
North Africa and Middle East	182,440 (92,260 to 306,269)	148.83 (75.39 to 249.68)	262,207 (133,613 to 435,252)	150.19 (76.58 to 249.3)	0.02% (0.01 to 0.02)
Oceania	2,099 (963 to 3,665)	90.59 (41.62 to 158.16)	4,181 (1,974 to 7,213)	95.54 (45.18 to 164.75)	0.18% (0.17 to 0.19)
South Asia	878,285 (455,047 to 1,451,234)	236.4 (122.64 to 390.16)	13,73,374 (715,575 to 2,243,658)	250.36 (130.24 to 409.52)	0.28% (0.22 to 0.33)
Southeast Asia	1,75,299 (81,995 to 305,432)	108.73 (50.87 to 189.5)	1,78,053 (87,707 to 301,496)	100.42 (49.38 to 170.16)	−0.41% (−0.5 to −0.32)
Southern Latin America	23,076 (11,594 to 38,844)	160.59 (80.64 to 270.39)	27,549 (15,207 to 44,642)	174.71 (96.3 to 283.19)	0.17% (0.06 to 0.28)
Southern Sub-Saharan Africa	35,319 (18,175 to 58,040)	186.99 (96.26 to 307.2)	45,090 (23,296 to 74,634)	192.99 (99.69 to 319.59)	0.12% (0.11 to 0.14)
Tropical Latin America	41,385 (20,694 to 69,119)	79.79 (39.94 to 133.33)	62,944 (34,125 to 102,260)	122.87 (66.42 to 199.7)	1.12% (0.65 to 1.59)
Western Europe	69,791 (32,493 to 120,841)	84.68 (39.07 to 146.67)	62,929 (31,789 to 105,681)	84.98 (42.73 to 142.69)	0.31% (0.19 to 0.44)
Western Sub-Saharan Africa	95,415 (47,729 to 160,989)	139.28 (69.93 to 234.63)	2,59,094 (130,721 to 434,845)	141.22 (71.43 to 236.89)	0.05% (0.04 to 0.06)

**Table 2:** Deaths of pediatric stone disease in 1990 and 2021 for both sexes and all locations, with EAPC from 1990 to 2021.

location	Num_1990	ASR_1990	Num_2021	ASR_2021	EAPC_CI
Andean Latin America	0 (0 to 0)	0 (0 to 0)	0 (0 to 0)	0 (0 to 0)	−0.18% (−0.55 to 0.19)
Australasia	0 (0 to 0)	0 (0 to 0)	0 (0 to 0)	0 (0 to 0)	−2.01% (−2.7 to −1.31)
Caribbean	0 (0 to 0)	0 (0 to 0)	0 (0 to 0)	0 (0 to 0)	1.3% (1.07 to 1.53)
Central Asia	1 (1 to 2)	0.01 (0 to 0.01)	1 (1 to 1)	0 (0 to 0.01)	−1.49% (−1.73 to −1.24)
Central Europe	2 (1 to 2)	0.01 (0 to 0.01)	0 (0 to 0)	0 (0 to 0)	−6.72% (−7.59 to −5.83)
Central Latin America	4 (4 to 5)	0.01 (0.01 to 0.01)	3 (2 to 3)	0 (0 to 0)	−1.2% (−1.39 to −1.01)
Central Sub-Saharan Africa	0 (0 to 1)	0 (0 to 0)	1 (0 to 1)	0 (0 to 0)	−1.18% (−1.28 to −1.08)
East Asia	44 (13 to 65)	0.01 (0 to 0.02)	5 (2 to 8)	0 (0 to 0)	−6.93% (−7.18 to −6.68)
Eastern Europe	3 (3 to 4)	0.01 (0.01 to 0.01)	1 (1 to 1)	0 (0 to 0)	−2.3% (−2.79 to −1.8)
Eastern Sub-Saharan Africa	7 (2 to 16)	0.01 (0 to 0.02)	10 (5 to 25)	0.01 (0 to 0.02)	−1.33% (−1.39 to −1.27)
Global	120 (56 to 165)	0.01 (0 to 0.01)	66 (43 to 94)	0 (0 to 0)	−2.5% (−2.59 to −2.41)
High-income Asia Pacific	0 (0 to 0)	0 (0 to 0)	0 (0 to 0)	0 (0 to 0)	0.14% (−0.24 to 0.53)
High-income North America	1 (1 to 1)	0 (0 to 0)	1 (1 to 1)	0 (0 to 0)	0.95% (0.52 to 1.38)
High-middle SDI	17 (10 to 23)	0.01 (0 to 0.01)	4 (3 to 5)	0 (0 to 0)	−4.65% (−4.89 to −4.41)
High SDI	3 (3 to 4)	0 (0 to 0)	2 (2 to 2)	0 (0 to 0)	−1.13% (−1.52 to −0.74)
Low-middle SDI	27 (9 to 46)	0.01 (0 to 0.01)	23 (13 to 33)	0 (0 to 0.01)	−1.52% (−1.64 to −1.4)
Low SDI	13 (5 to 25)	0.01 (0 to 0.01)	17 (10 to 34)	0 (0 to 0.01)	−1.62% (−1.68 to −1.57)
Middle SDI	59 (23 to 82)	0.01 (0 to 0.01)	20 (11 to 27)	0 (0 to 0)	−3.48% (−3.62 to −3.33)
North Africa and Middle East	12 (4 to 18)	0.01 (0 to 0.01)	7 (2 to 11)	0 (0 to 0.01)	−2.22% (−2.57 to −1.87)
Oceania	0 (0 to 0)	0 (0 to 0)	0 (0 to 0)	0 (0 to 0)	−1.56% (−1.69 to −1.42)
South Asia	26 (6 to 46)	0.01 (0 to 0.01)	16 (7 to 25)	0 (0 to 0)	−2.63% (−2.78 to −2.47)
Southeast Asia	13 (5 to 20)	0.01 (0 to 0.01)	9 (3 to 13)	0.01 (0 to 0.01)	−1.47% (−1.57 to −1.37)
Southern Latin America	0 (0 to 0)	0 (0 to 0)	0 (0 to 0)	0 (0 to 0)	1.38% (0.59 to 2.18)
Southern Sub-Saharan Africa	0 (0 to 1)	0 (0 to 0)	1 (0 to 1)	0 (0 to 0)	0.45% (−0.02 to 0.93)
Tropical Latin America	2 (1 to 2)	0 (0 to 0)	4 (4 to 5)	0.01 (0.01 to 0.01)	4.1% (3.78 to 4.43)
Western Europe	1 (1 to 1)	0 (0 to 0)	1 (1 to 1)	0 (0 to 0)	−1.33% (−1.5 to −1.16)
Western Sub-Saharan Africa	4 (2 to 7)	0.01 (0 to 0.01)	7 (4 to 13)	0 (0 to 0.01)	−0.92% (−1.01 to −0.82)

**Table 3:** DALYs of pediatric stone disease in 1990 and 2021 for both sexes and all locations, with EAPC from 1990 to 2021.

location	Num_1990	ASR_1990	Num_2021	ASR_2021	EAPC_CI
Andean Latin America	60 (33 to 100)	0.45 (0.24 to 0.74)	81 (46 to 134)	0.46 (0.26 to 0.75)	0.23% (0.17 to 0.29)
Australasia	11 (5 to 20)	0.22 (0.1 to 0.4)	12 (5 to 22)	0.2 (0.09 to 0.37)	−0.22% (−0.3 to −0.14)
Caribbean	45 (22 to 79)	0.4 (0.2 to 0.7)	48 (25 to 84)	0.42 (0.21 to 0.72)	0.17% (0.15 to 0.19)
Central Asia	327 (194 to 498)	1.49 (0.88 to 2.27)	315 (189 to 502)	1.3 (0.77 to 2.08)	−0.42% (−0.49 to −0.34)
Central Europe	446 (279 to 694)	1.44 (0.9 to 2.24)	179 (97 to 302)	0.97 (0.53 to 1.64)	−1.3% (−1.46 to −1.14)
Central Latin America	559 (424 to 766)	0.94 (0.71 to 1.29)	453 (319 to 670)	0.68 (0.47 to 1)	−0.78% (−1 to −0.56)
Central Sub-Saharan Africa	101 (52 to 175)	0.5 (0.26 to 0.87)	238 (129 to 399)	0.47 (0.25 to 0.78)	−0.21% (−0.23 to −0.19)
East Asia	4,363 (1,902 to 6,305)	1.24 (0.53 to 1.79)	751 (464 to 1,157)	0.29 (0.18 to 0.44)	−5.32% (−5.58 to −5.06)
Eastern Europe	813 (531 to 1,246)	1.61 (1.05 to 2.47)	467 (273 to 766)	1.31 (0.77 to 2.15)	−0.65% (−0.78 to −0.52)
Eastern Sub-Saharan Africa	814 (392 to 1,548)	1.14 (0.55 to 2.16)	1,479 (871 to 2,729)	0.91 (0.54 to 1.69)	−0.78% (−0.83 to −0.74)
Global	16,751 (10,451 to 24,049)	1.02 (0.63 to 1.46)	14,230 (9,264 to 21,569)	0.71 (0.46 to 1.08)	−1.17% (−1.25 to −1.1)
High-income Asia Pacific	199 (103 to 346)	0.46 (0.24 to 0.8)	98 (54 to 163)	0.39 (0.21 to 0.64)	−0.87% (−1.08 to −0.67)
High-income North America	323 (186 to 538)	0.52 (0.3 to 0.86)	346 (225 to 540)	0.47 (0.31 to 0.73)	−0.86% (−1.32 to −0.39)
High-middle SDI	2,707 (1,799 to 3,933)	0.95 (0.63 to 1.37)	1,260 (754 to 2,035)	0.54 (0.32 to 0.87)	−2.14% (−2.33 to −1.96)
High SDI	1,029 (611 to 1,650)	0.52 (0.31 to 0.83)	757 (457 to 1,212)	0.41 (0.24 to 0.65)	−1.05% (−1.27 to −0.83)
Low-middle SDI	4,304 (2,322 to 6,711)	1.04 (0.57 to 1.62)	4,966 (3,075 to 7,656)	0.85 (0.53 to 1.31)	−0.59% (−0.63 to −0.54)
Low SDI	1,840 (984 to 2,941)	1 (0.54 to 1.61)	3,304 (2,055 to 5,197)	0.8 (0.5 to 1.26)	−0.77% (−0.8 to −0.74)
Middle SDI	6,858 (3,705 to 9,630)	1.2 (0.65 to 1.69)	3,934 (2,549 to 5,975)	0.68 (0.44 to 1.03)	−1.88% (−2 to −1.76)
North Africa and Middle East	1,455 (722 to 2,196)	1.15 (0.57 to 1.74)	1,311 (691 to 2,055)	0.75 (0.39 to 1.17)	−1.24% (−1.42 to −1.07)
Oceania	6 (3 to 11)	0.27 (0.12 to 0.48)	12 (5 to 22)	0.28 (0.12 to 0.51)	0.13% (0.12 to 0.14)
South Asia	4,474 (2,185 to 7,262)	1.17 (0.58 to 1.9)	5,013 (2,913 to 8,091)	0.92 (0.54 to 1.49)	−0.77% (−0.82 to −0.73)
Southeast Asia	1,485 (776 to 2,188)	0.92 (0.48 to 1.36)	1,197 (637 to 1,733)	0.67 (0.36 to 0.98)	−1.1% (−1.19 to −1.01)
Southern Latin America	68 (34 to 119)	0.47 (0.24 to 0.83)	83 (46 to 138)	0.53 (0.29 to 0.88)	0.27% (0.18 to 0.36)
Southern Sub-Saharan Africa	128 (72 to 209)	0.67 (0.38 to 1.11)	169 (99 to 270)	0.72 (0.43 to 1.16)	0.18% (0.05 to 0.3)
Tropical Latin America	236 (172 to 333)	0.46 (0.33 to 0.64)	497 (398 to 637)	0.96 (0.77 to 1.24)	2.79% (2.47 to 3.11)
Western Europe	280 (174 to 438)	0.34 (0.21 to 0.54)	219 (130 to 358)	0.3 (0.18 to 0.49)	−0.15% (−0.26 to −0.04)
Western Sub-Saharan Africa	556 (354 to 878)	0.79 (0.5 to 1.25)	1,262 (798 to 1,970)	0.68 (0.43 to 1.06)	−0.41% (−0.45 to −0.37)

### 3.2 Regional burden of pediatric stone disease

In the five SDI regions, the incidence of pediatric stone disease decreased over time in the high and high-middle SDI regions, whereas it increased in the middle, low-middle, and low SDI regions. Regarding mortality and DALYs, the low SDI region experienced an increase over time, while the other four regions saw a decline. In 2021, the low-middle SDI region had the highest burden, with 1,157,622 cases (95% UI: 597,885 to 1,896,931), 23 deaths (95% UI: 13 to 33), and 4,966 DALYs (95% UI: 3,075 to 7,656). We further analyzed ASIR, ASMR, and ASDR across the five SDI regions. Over time, ASIR decreased in the high, high-middle, and low SDI regions but increased in the middle and low-middle SDI regions. In contrast, ASMR and ASDR declined across all five SDI regions. In 2021, the low-middle SDI regions reported the highest ASIR and ASDR at 197.65 per 100,000 people and 0.85 per 100,000 people respectively, but all five SDI regions had zero ASMR. The low-middle SDI region showed the largest increase in ASIR, with an EAPC of 0.18% (95% CI: 0.15 to 0.2). In contrast, ASMR and ASDR declined the most in the high-middle SDI regions, with EAPC of −4.65% (95% CI: −4.89 to −4.41) and −2.14% (95% CI: −2.33 to −1.96), respectively (Tables 1–3, Figures 1A,B).

At the regional level, according to the GBD classification, South Asia had the highest incidence, mortality, and DALYs for pediatric stone disease in 2021, with 1,373,374 cases (95% UI: 715,575 to 2,243,658), 16 deaths (95% UI: 7 to 25), and 5,031 DALYs (95% UI: 2,913 to 8,091). Eastern Europe had the highest ASIR and ASDR at 386.64 and 1.31 per 100,000 people, respectively, while all regions of the world had an ASMR of 0. From 1990 to 2021, Tropical Latin America showed the most significant increase in ASIR (EAPC = 1.12%, 95% CI: 0.65 to 1.59), ASMR (EAPC = 4.1%, 95% CI: 3.78 to 4.43), and ASDR (EAPC = 2.79%, 95% CI: 2.47 to 3.11). In contrast, East Asia experienced the steepest decline, with ASIR decreasing by an EAPC of −2.16% (95% CI: −2.48 to −1.83), ASMR decreasing by an EAPC of −6.93% (95% CI: −7.18 to −6.68) and ASDR decreasing by an EAPC of −5.32% (95% CI: −5.58 to −5.06) (Tables 1–3).

### 3.3 National burden of pediatric stone disease

**Figure 2:**
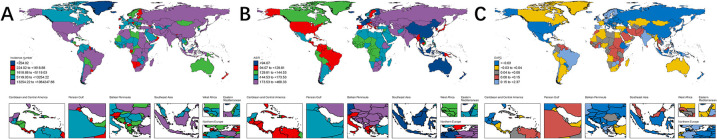
Global burden of pediatric stone disease in 204 countries and regions. Number of cases of pediatric stone disease in 2021 **(A)**; ASIR of pediatric stone disease in 2021 **(B)**; EAPC of ASIR of pediatric stone disease from 1990 to 2021 **(C)**.

Nationally, India reported the highest incidence, mortality, and DALYs of pediatric stone disease in 2021, with 1,064,248 cases (95% UI: 554,992–1,740,564), 9 deaths (95% UI: 4–15), and 3,638 DALYs (95% UI: 2,069–5,951). Regarding age-standardized rates, Armenia had the highest ASIR, at 468.33 per 100,000. Twenty-eight countries, including Afghanistan, Armenia, and Brazil, recorded the highest ASMR, at 0.01 per 100,000. Armenia and Kazakhstan reported the highest ASDR, both at 1.75 per 100,000. Greece exhibited the most significant increase in ASIR, with an EAPC of 1.99% (95% CI: 1.71 to 2.26). Kuwait showed the largest rise in ASMR, with an EAPC of 20.66% (95% CI: 17.36 to 24.05). Brazil experienced the most significant increase in ASDR, with an EAPC of 2.83% (95% CI: 2.5 to 3.15) (Supplementary Tables S1–S3, Figures 2A–C).

### 3.4 Global burden of pediatric stone disease by age and region

**Figure 3:**
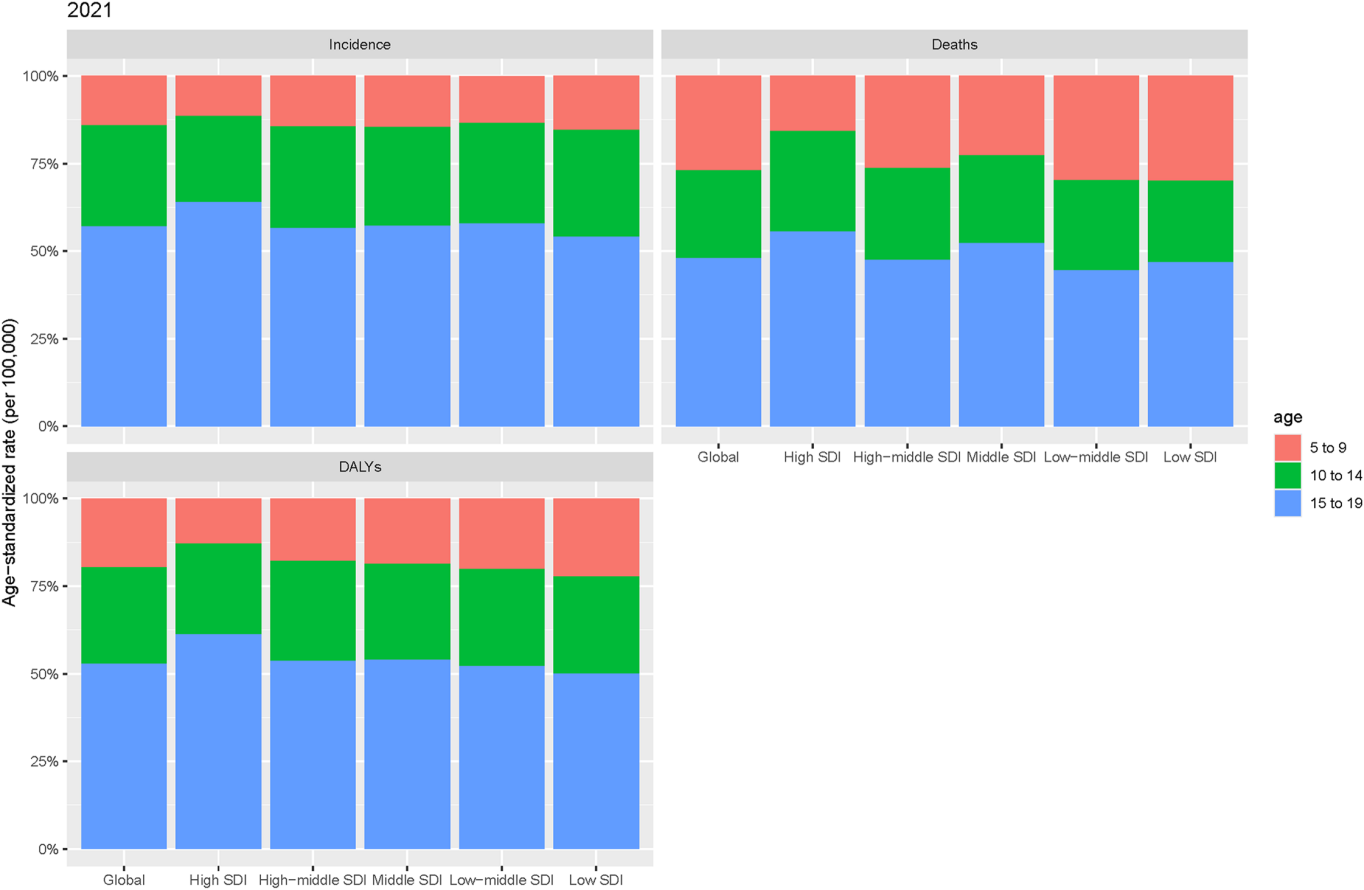
Age-specific incidence, mortality, and DALYs for pediatric stone disease by region in 2021.

A detailed age-based subgroup analysis in 2021 revealed significant differences in the burden of pediatric stone disease, both globally and across the five SDI regions. A clear pattern emerged when comparing the different age groups. In the 5–9 years age group, ASIR and ASDR for pediatric stone disease were the lowest globally and within the five SDI regions. The ASMR in this age group was lowest in the high and middle SDI regions, matching the 10–14 years age group globally and in the high-middle SDI regions. However, in the low-middle and low SDI regions, the ASMR for the 5–9 years age group exceeded that of the 10–14 years age group. In contrast, the 15–19 years age group exhibited the highest ASR for pediatric kidney stones, both globally and across all five SDI regions. In summary, children aged 15–19 years bear the greatest burden of pediatric stone disease (Figure 3).

**Figure 4:**
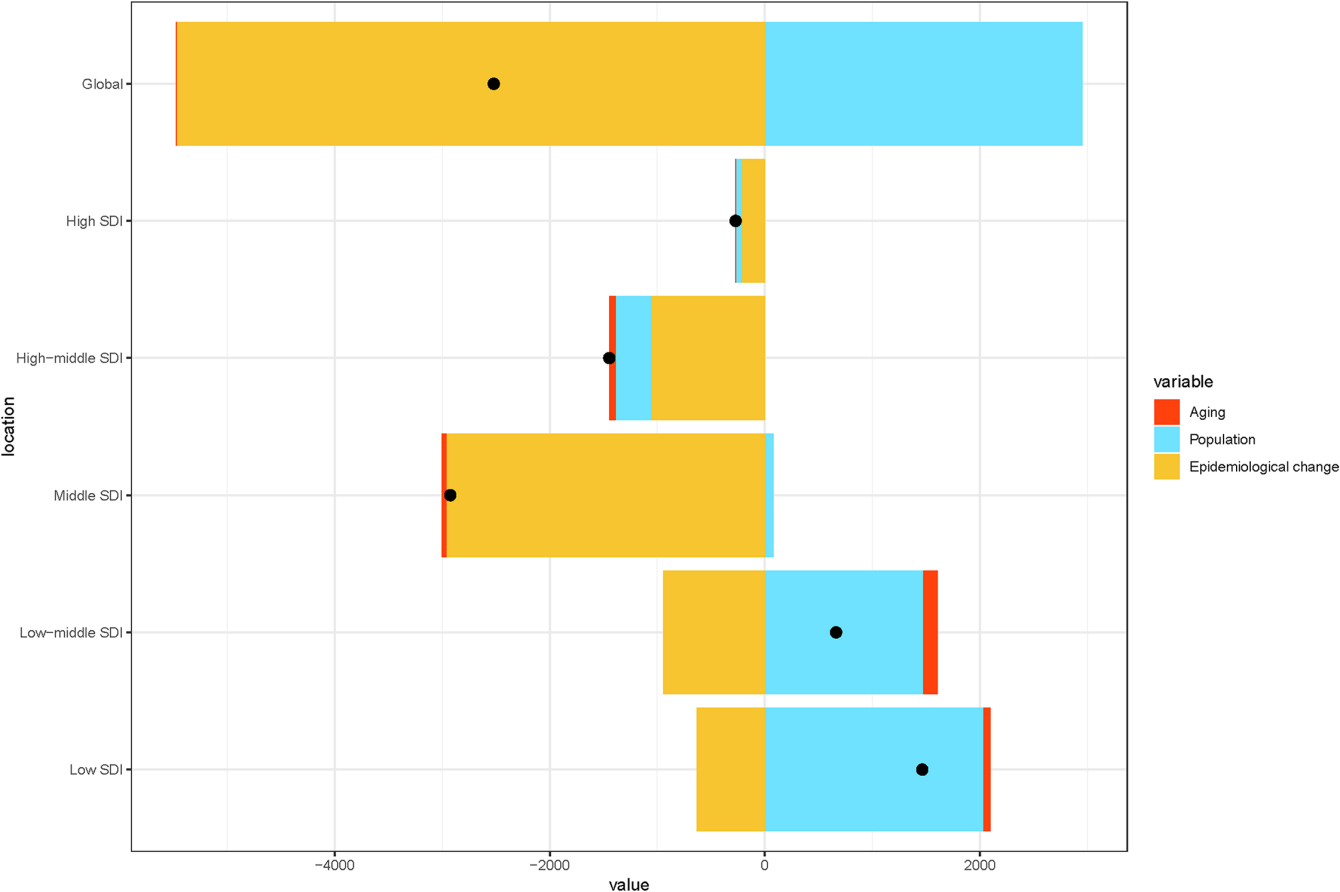
Changes in pediatric stone disease globally and in five SDI regions from 1990 to 2021, based on population-level determinants of population growth, aging, and epidemiological changes. The black dots represent the total values of the changes contributed by all three components.

We also analyzed changes in the burden of kidney stones in children globally and within the five SDI regions from 1990 to 2021, focusing on population-level determinants, such as population growth, aging, and epidemiological shifts. Over the past 31 years, epidemiological changes have been the primary driver of the global increase in pediatric kidney stone burden, especially in the high, high-middle, and middle SDI regions. In contrast, population growth emerged as the main factor influencing the burden of pediatric kidney stones in the low-middle and low-SDI regions. Aging had a minimal impact on the burden of kidney stones in children both globally and within the five SDI regions (Figure 4).

### 3.5 The association between SDI, global inequalities, and the burden of pediatric stone disease

**Figure 5:**
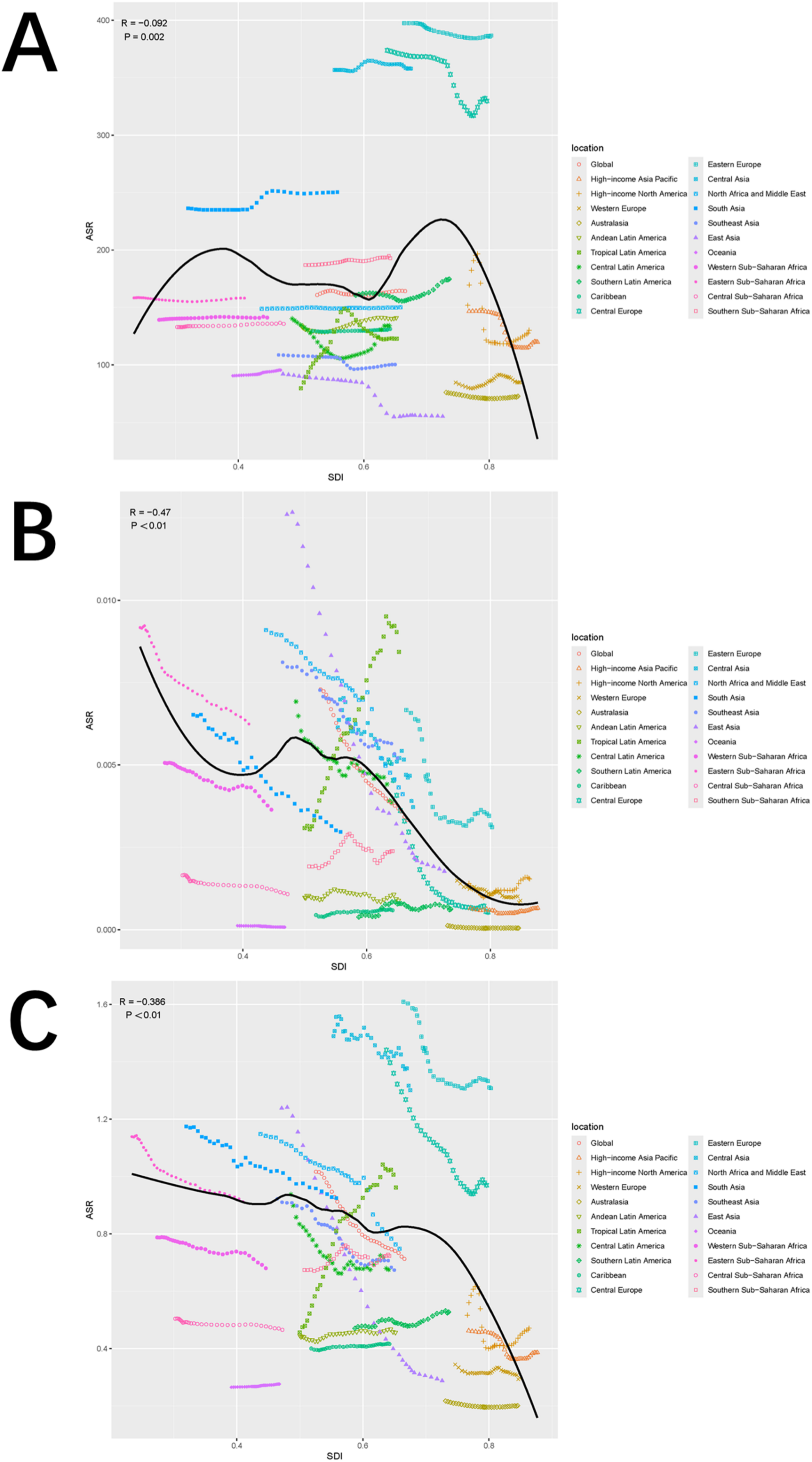
Trends in ASIR **(A)**, ASMR **(B)**, and ASDR **(C)** of pediatric stone disease across 21 geographical regions and globally by SDI for both sexes combined for 1990–2021. For each region, the points from left to right depict the estimates from 1990 to 2021. The shaded areas represent 95% UI.

**Figure 6:**
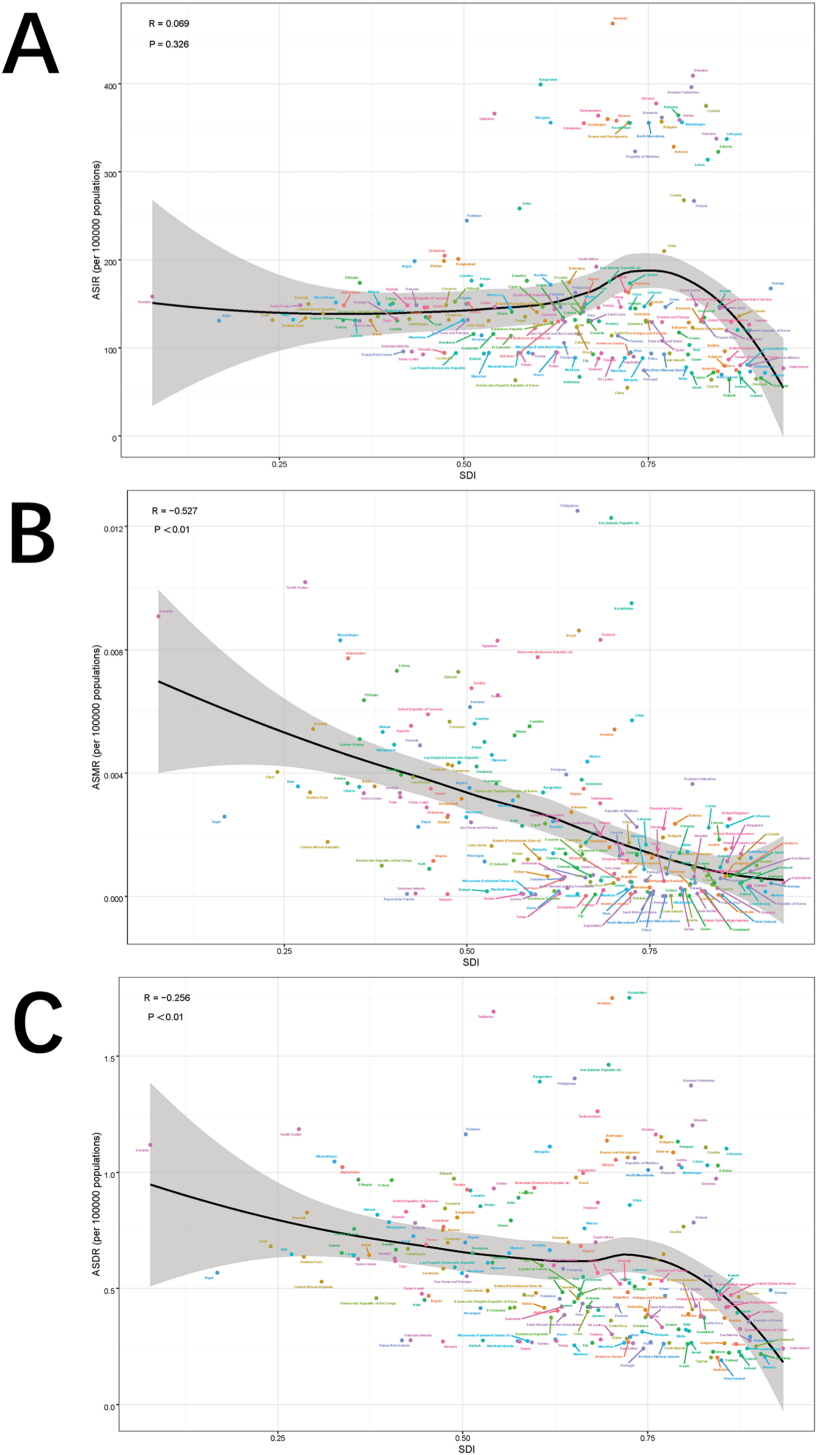
Correlation between ASIR and SDI quintile **(A)**, correlation between ASMR and SDI quintile **(B)**, and correlation between age-specific DALYs rate and SDI quintile **(C)**. The ρ and *p* values were derived from Pearson correlation analysis.

**Figure 7:**
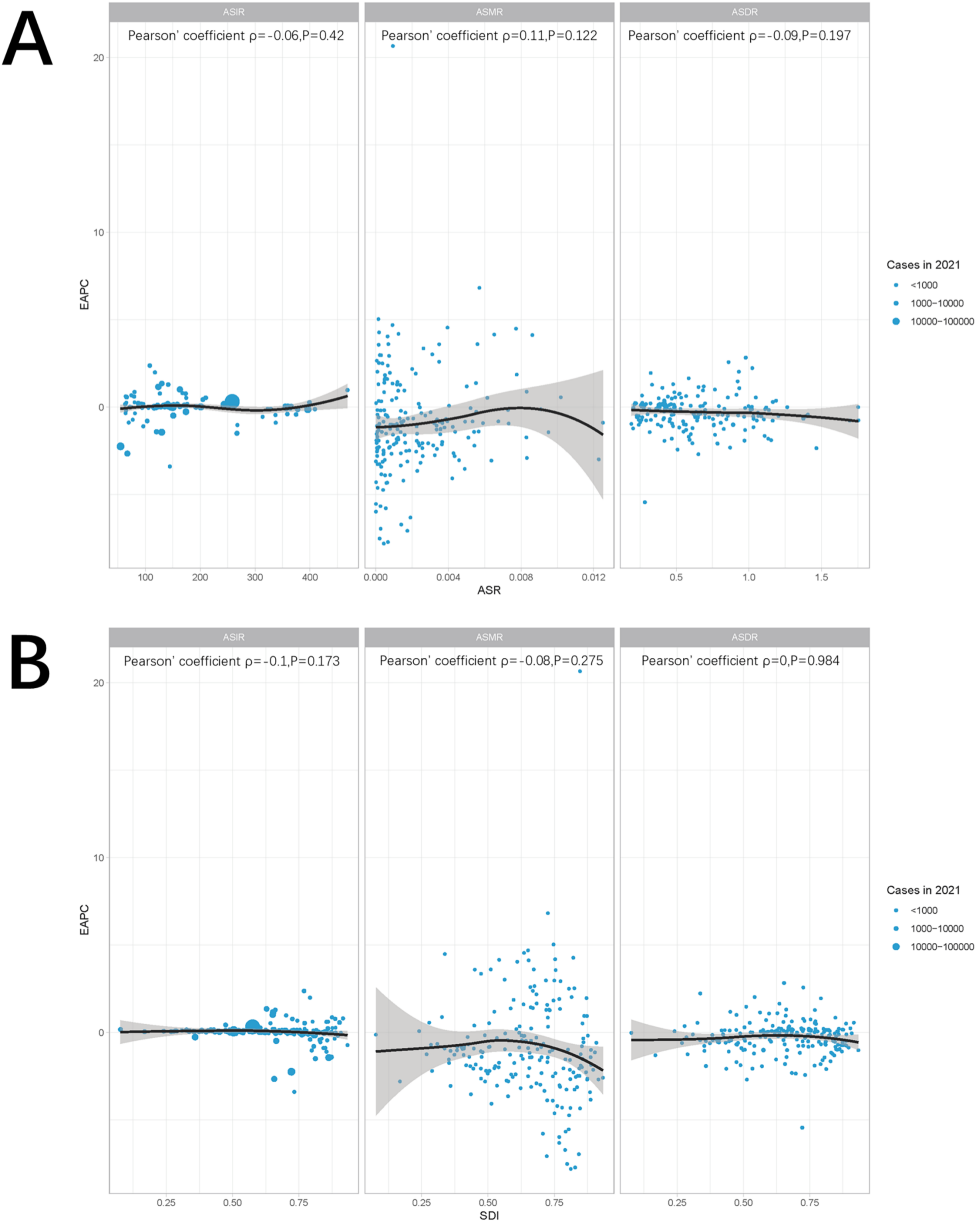
Correlation between ASR and EAPC **(A)** and SDI and EAPC **(B)**. The ρ and *p* values were derived from Pearson correlation analysis.

Globally and across 21 regions, the ASIR of pediatric stone disease initially increases, then decreases, and repeats this cycle with rising SDI levels. ASIR peaks at an SDI of approximately 0.72, and reaches its minimum when the SDI exceeds 0.8. Both ASMR and ASDR exhibited a general downward trend as SDI increased. However, slight increases in ASMR are observed at SDI ranges of 0.4–0.48 and 0.55–0.57, and ASDR shows minor increases at SDI levels of 0.45–0.48 and 0.61–0.65. Both ASMR and ASDR reach their highest values when SDI is below 0.3, and their lowest values when SDI exceeds 0.8. This pattern suggests that areas with lower SDI levels experience a higher burden of pediatric stone disease (Figures 5A–C). In 2021, an analysis of 204 countries found that ASMR (*R* = −0.527, *p* < 0.01) and ASDR (*R* = −0.256, *p* < 0.01) in children with kidney stones were negatively correlated with SDI, indicating that ASMR and ASDR decrease as SDI increases. However, no statistically significant correlation was found between ASIR and SDI (Figures 6A–C). Further analysis examined the relationship between EAPCs and ASR metrics in 2021 relative to the SDI by country or region. As ASMR and ASDR increased, their respective EAPCs showed a declining trend. Conversely, higher ASIR values are generally associated with increasing EAPCs for ASIR. However, the correlation between the ASR values and their EAPCs was not statistically significant. Additionally, the EAPCs of ASR exhibited a downward trend as SDI increased, although no significant relationship was observed between the EAPCs of ASR and their associated EAPCs (Figures 7A, B).

### 3.6 Predictions of pediatric stone disease

We applied the BAPC model to analyze ASIR, ASMR, and ASDR for the burden of pediatric stone disease.

**Figure 8:**
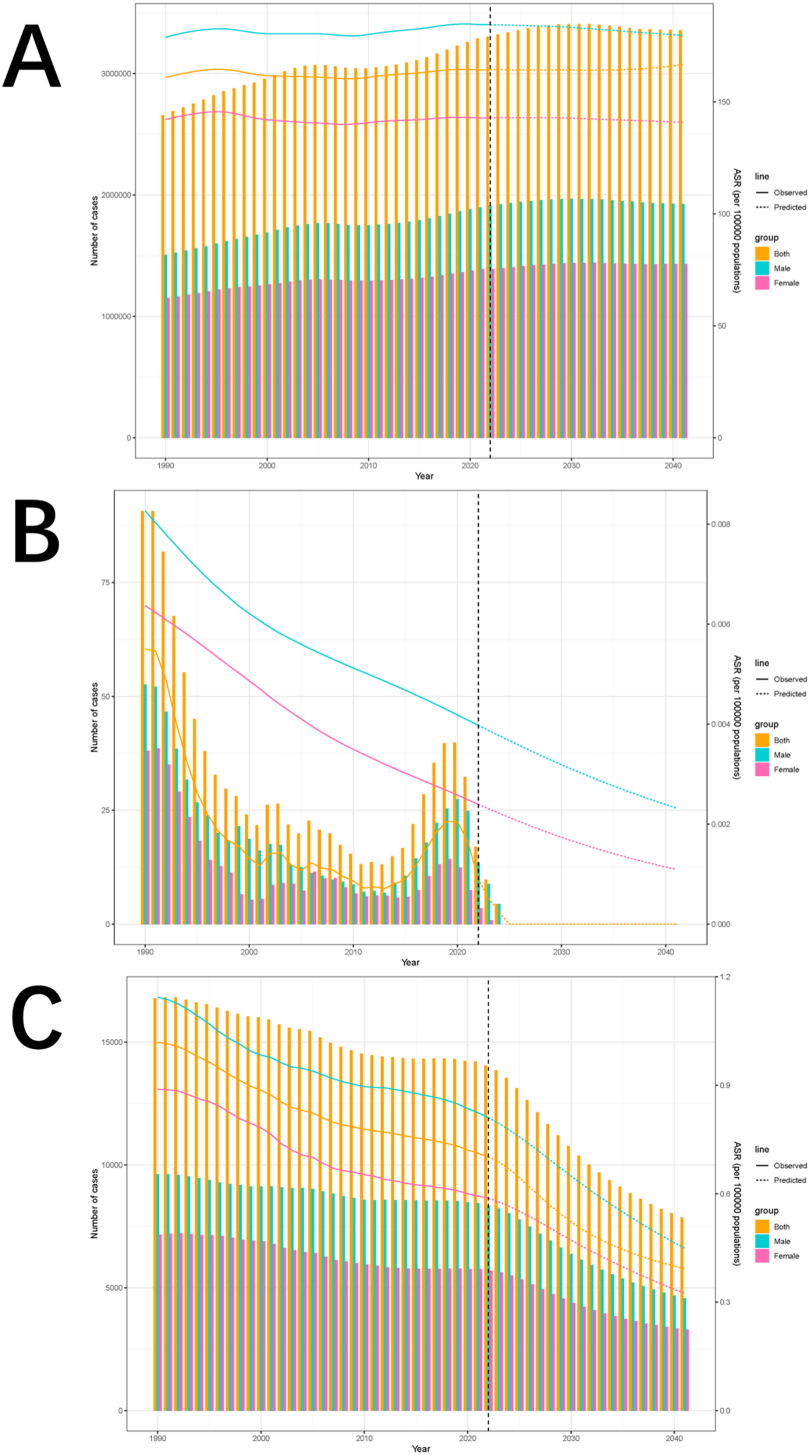
Incidence **(A)**, deaths **(B)**, and DALYs trends of pediatric stone disease from 1990 to 2021 and projections from 2022 to 2041 **(C)**.

Globally, the ASIR for both boys and girls are projected to decline from 2022 to 2041. For boys, ASIR is expected to decrease from 184.47 per 100,000 in 2022 (95% UI: 179.97 to 188.98) to 179.69 per 100,000 in 2041 (95% UI: 92.42 to 266.95). For girls, ASIR is projected to decline from 142.89 per 100,000 in 2022 (95% UI: 139.01 to 146.78) to 140.96 per 100,000 in 2041 (95% UI: 65.8 to 216.13). ASMR and ASDR are also expected to decrease for both sexes over the same period, with ASMR likely to remain at zero for boys and girls throughout the next two decades. In boys, ASDR is projected to decline from 0.81 per 100,000 in 2022 (95% UI: 0.78 to 0.84) to 0.45 per 100,000 in 2041 (95% UI: 0.13 to 0.77). For girls, ASDR is expected to drop from 0.59 per 100,000 (95% UI: 0.55 to 0.62) in 2022 to 0.32 per 100,000 (95% UI: 0.01 to 0.64) by 2041. These projections emphasize significant gender differences, with the ASR consistently higher in boys than in girls throughout the projected period. (Figures 8A–C; Supplementary Table S4).

We also analyzed sex-disaggregated projections of pediatric stone disease incidence, mortality, and DALYs from 2022 to 2041. The incidence is expected to increase in both boys and girls. In boys, the incidence is projected to increase from 1,914,477 cases (95% UI: 1,508,791 to 2,320,163) by 2022 to 1,925,573 cases (95% UI: 364,174 to 3,597,063) by 2041. Among girls, the incidence is expected to increase from 1,390,578 cases (95% UI: 990,968 to 1,790,188) in 2022 to 1,432,699 cases (95% UI: 133,360 to 2,987,354) in 2041. In contrast, mortality is expected to decline in both sexes. In boys, mortality is projected to decrease from 14 deaths in 2022 (95% UI: 0 to 1,354,231) to 0 deaths in 2041 (95% UI: 0 to 10,894,617). For girls, mortality is expected to decrease from 4 deaths in 2022 (95% UI: 0 to 1,573,542) to 0 deaths in 2041 (95% UI: 0 to 1,040,326). DALYs are expected to follow the same downward trend as mortality. In boys, DALYs are projected to decline from 8,359 (95% UI: 0–740,081) in 2022 to 4,569 (95% UI: 0–3,663,503) in 2041. For girls, DALYs are expected to drop from 5,701 cases (95% UI: 0–854,576) in 2022 to 3,298 cases (95% UI: 0–3,152,829) in 2041.Notably, incidence, mortality, and DALYs were consistently lower in girls than in boys throughout the forecasted period (Figures 8A–C, Supplementary Table S5).

## 4 Discussion

The prevalence of the Urinary stone disease among children has increased from 4 to 6% to 10% in recent years (2, 25, 26). This increase presents complex challenges for global public health, driving ongoing discussions about its worldwide burden (27–29). In this study, we analyzed the disease burden across diverse populations and time periods using the latest GBD database. Additionally, we evaluated epidemiological trends in pediatric stone disease over the past 31 years using EAPC calculations. However, this study has several limitations that warrant consideration.

From 1990 to 2021, the global incidence of pediatric stone disease among young people has increased significantly from 2,659,391 to 3,289,663 cases. However, the number of deaths and DALYs associated with the disease decreased during the same period. Similarly, ASIR, ASMR, and ASDR followed this trend, with a rising incidence, but declining mortality and DALYs. This pattern may be attributed to improvements in the diagnosis and management of pediatric stone disease, leading to better clinical outcomes and reduced disease burden.

In our demographic analysis, children aged 15–19 years bore the greatest burden of pediatric stone disease globally and across all five SDI regions, with the highest ASR. Although numerous predisposing factors contribute to pediatric stone disease, many can be mitigated or even avoided, making the condition largely preventable. Various factors influence stone formation, including the concentration of stone-forming ions in urine, urine pH, flow rate, metabolic crystallization, and anatomical considerations (3–6, 26). Early European pediatric studies identified infections as the primary cause of urolithiasis (7, 17). However, recent trends have revealed that metabolic factors dominate pediatric urolithiasis cases (2, 30, 31), with most pediatric stone cases being linked to metabolic issues (5, 6, 8, 30, 31). Hypercalciuria is a key metabolic risk factor, contributing not only to stone formation but also to recurrent urinary tract infections and decreased bone mineral density in children (5, 12). Hyperoxaluria also emerges as a significant risk factor for stone formation in this population (11, 16). Anatomical abnormalities, such as ureterocele, further elevate the risk by causing urinary retention, which promotes infections and exacerbates stone formation (11, 16, 32). Nutritional factors also play a crucial role. A protein-rich diet increases uric acid, calcium, and oxalate levels, thereby increasing the risk of stone formation (33). Other contributing factors include a ketogenic diet, high-fat diet, and obesity, all of which are linked to an increased risk of stone formation (34, 35). Additionally, conditions such as inflammatory bowel disease, cystic fibrosis, prematurity, long-term immobilization, and the use of antiepileptic medications (e.g., topiramate) are associated with urinary stone disease in children (8). To reduce the disease burden, treatment priorities include increasing fluid intake and limiting excessive salt consumption. Drug therapy is tailored to address underlying metabolic disorders (36). Surgical intervention is required when ureteral stones fail to pass naturally or when kidney stones become symptomatic, with surgery necessary in up to 22% of acute pediatric stone episodes (37). SWL is an effective treatment option for pediatric stone disease (9). SWL is minimally invasive and achieves a high stone clearance rate (10). However, complications such as intestinal perforation, renal colic, and hematuria can occur (13, 14, 38).

Regional differences in the burden of pediatric stone disease underscore the importance of identifying hotspots to effectively target at-risk populations. In 2021, according to the GBD classification, South Asia reported the highest incidence, mortality, and DALYs, likely because of its large population base and widespread implementation of disease screening. Eastern Europe has the highest ASIR and ASDR, while ASMR is 0 in all regions of the world. These variations highlight the influence of socioeconomic factors, such as lifestyle, healthcare access, and environmental exposures, on health outcomes. We also analyzed the association between the five SDI regions and ASIR, ASMR, and ASDR in pediatric stone disease. In 2021, the low-middle SDI region had the highest ASIR. This finding reflects both advancements in diagnostic techniques and instruments for pediatric lithiasis in the region and a potential shortage of medical resources (39). Across all five SDI regions, ASMR and ASDR declined over time, suggesting more effective health interventions, increased disease awareness, and better treatment outcomes. The disparity in ASR between regions with varying SDI levels may be attributed to differences in environmental factors, lifestyle, and disease control strategies (36). These trends illustrate the complex relationship between socioeconomic development and disease burden, emphasizing the need for targeted public health strategies and resource allocation. Tailored efforts are essential to mitigate pediatric stone disease effectively, ensuring that healthcare resources align with regional needs and challenges (40).

Based on the GBD 2021 study, this study projects the global burden of pediatric stone disease from 2022 to 2041. While the ASR for the disease is expected to decline, the incidence is projected to increase, with mortality and DALYs following the same downward trend. Additionally, the burden of pediatric stone disease was anticipated to remain higher in boys than in girls throughout the study period. Managing the health challenges posed by pediatric stone diseases will continue to be a global public health priority. Addressing this burden will require coordinated efforts to control high-risk factors, enhance preventive strategies, and improve care and health promotion. These efforts must focus on both clinical management and broader public health initiatives to effectively reduce the impact of the disease.

Our study, while providing valuable insights, has several limitations that should be acknowledged. First, clinical processes, such as data collection and patient care for pediatric stone disease during 2020–2021, may have been disrupted by the COVID-19 pandemic. Although the GBD research process attempts to mitigate these limitations through various methods, including modeling to estimate missing data and adjusting analytical strategies to account for the pandemic's influence (41), these adjustments may introduce additional uncertainty. Moreover, while the GBD database covers many countries and regions globally, the accuracy and completeness of the data vary across regions. This issue is particularly relevant in low- and middle-income countries, where the data may be less detailed or subject to bias. Additionally, differences in disease classifications and definitions between the GBD database and other international databases or studies may affect the precision of burden assessment. Variations in medical practices, diagnostic techniques, and reporting systems across regions and periods can also introduce bias, complicating the analysis of long-term trends. Furthermore, although the GBD database employs multiple epidemiological indicators to comprehensively assess disease burden, these metrics may not fully capture the impact of pediatric stone disease on patients' quality of life and socioeconomic status. Finally, because the GBD database provides population-level data without detailed individual patient information, the ability to conduct in-depth analyses of the epidemiological characteristics of pediatric stone disease is limited.

## 5 Conclusion

In conclusion, our study indicates that the burden of pediatric stone disease is highest among individuals aged 15–19 years, boys, and those residing in the low-middle SDI regions. Although the global burden of the disease has declined from 1990 to 2021, it remains a significant public health concern. Addressing this burden requires urgent action and coordinated efforts to enhance early detection and treatment strategies. Our projections suggest that by 2041, the ASR will decline for both boys and girls. However, sex-stratified projections indicate that the incidence will continue to rise for both sexes, while mortality and DALYs are expected to decline. We hope that these findings will offer valuable insights to policymakers, guiding the development of more effective disease control measures and prevention strategies.

## Data Availability

The original contributions presented in the study are included in the article/Supplementary Material, further inquiries can be directed to the corresponding author/s.
