# Supplementary material for: The global, regional, and national burden of pediatric stone disease: 1990–2021 and projections for the next two decades
Source: Front Pediatr. 2025 Mar 25;13:1529407. doi: 10.3389/fped.2025.1529407 (PMC11975870; doi:10.3389/fped.2025.1529407)
Supplement: Supplementary file 1 [file Table1.docx]

**Supplementary Material**

***Table S1. pediatric stone disease incidence for both sexes and for 204 countries in 1990 and 2021, and estimated annual percentage changes from 1990 to 2021.***

| location | Num_1990 | ASR_1990 | Num_2021 | ASR_2021 | EAPC_CI |
| --- | --- | --- | --- | --- | --- |
| Afghanistan | 5651 (2749 to 9681) | 142.96 (69.44 to 244.96) | 17786 (8876 to 29917) | 148.27 (74.09 to 249.2) | 0.09% (0.07 to 0.1) |
| Albania | 3833 (2083 to 6217) | 365.62 (198.65 to 592.87) | 1799 (972 to 2888) | 357.92 (193.28 to 574.56) | -0.08% (-0.09 to -0.07) |
| Algeria | 13668 (6790 to 23308) | 143.88 (71.59 to 245.26) | 16216 (8131 to 27208) | 145.18 (73.15 to 243.31) | 0.03% (0.02 to 0.03) |
| American Samoa | 14 (7 to 25) | 88.84 (40.9 to 155.5) | 15 (7 to 25) | 93.39 (43.46 to 161.59) | 0.14% (0.13 to 0.15) |
| Andorra | 9 (4 to 16) | 75.29 (32.78 to 134.41) | 10 (4 to 17) | 74.81 (32.55 to 134.16) | 0% (-0.02 to 0.01) |
| Angola | 4914 (2445 to 8411) | 134.35 (67.19 to 229.68) | 16636 (8332 to 28016) | 135.11 (68.17 to 227.04) | 0.03% (0.02 to 0.05) |
| Antigua and Barbuda | 23 (11 to 39) | 127.45 (63.39 to 216.49) | 25 (12 to 43) | 129.7 (64.31 to 222.58) | 0.07% (0.06 to 0.07) |
| Argentina | 15275 (7566 to 25737) | 160.19 (79.4 to 269.93) | 18062 (8916 to 31072) | 162.89 (80.25 to 280.32) | 0.03% (0.02 to 0.05) |
| Armenia | 3561 (1905 to 5859) | 381.5 (203.98 to 627.44) | 2680 (1486 to 4231) | 468.33 (259.76 to 739.81) | 0.97% (0.8 to 1.15) |
| Australia | 3081 (1350 to 5524) | 72.86 (31.63 to 130.69) | 3530 (1546 to 6376) | 73.38 (32.09 to 132.56) | -0.01% (-0.01 to 0) |
| Austria | 2182 (1542 to 2966) | 137.95 (96.9 to 188.08) | 1362 (688 to 2256) | 96.14 (48.18 to 159.44) | -1.1% (-1.38 to -0.82) |
| Azerbaijan | 7949 (4270 to 13168) | 355.58 (190.87 to 588.7) | 8276 (4375 to 13591) | 359.71 (190.21 to 590.91) | 0.07% (0.06 to 0.08) |
| Bahamas | 108 (53 to 185) | 128.16 (62.8 to 219.85) | 130 (65 to 222) | 128.74 (64.36 to 220.32) | 0.05% (0.04 to 0.06) |
| Bahrain | 187 (93 to 315) | 142.84 (71.1 to 240.04) | 483 (242 to 797) | 149.53 (74.67 to 246.77) | 0.14% (0.11 to 0.17) |
| Bangladesh | 78867 (40048 to 131335) | 200.47 (102.04 to 333.29) | 96692 (49594 to 159007) | 200.97 (102.93 to 330.9) | 0.01% (0 to 0.02) |
| Barbados | 88 (43 to 150) | 129.11 (62.98 to 220.15) | 74 (37 to 125) | 131.16 (65.23 to 221.93) | 0.09% (0.07 to 0.1) |
| Belarus | 7963 (4170 to 13347) | 338.7 (177.29 to 567.61) | 4970 (2580 to 8388) | 328.34 (170.43 to 554.5) | -0.11% (-0.12 to -0.1) |
| Belgium | 1617 (744 to 2898) | 79.46 (36.25 to 142.56) | 1606 (709 to 2855) | 79.57 (34.97 to 141.49) | 0.61% (-0.16 to 1.37) |
| Belize | 91 (45 to 157) | 128.14 (63.41 to 220.92) | 180 (91 to 306) | 130.74 (65.58 to 222.73) | 0.06% (0.05 to 0.07) |
| Benin | 2208 (1077 to 3743) | 129.68 (63.79 to 218.87) | 6502 (3217 to 10997) | 131.05 (65.08 to 221.2) | 0.03% (0.02 to 0.04) |
| Bermuda | 15 (7 to 27) | 128.83 (61.94 to 222.6) | 12 (6 to 21) | 129.78 (63.88 to 221.05) | 0.04% (0.03 to 0.05) |
| Bhutan | 491 (246 to 844) | 197.07 (98.53 to 338.62) | 410 (206 to 697) | 198.71 (99.68 to 337.66) | 0.04% (0.03 to 0.04) |
| Bolivia (Plurinational State of) | 3156 (1546 to 5396) | 139.91 (68.7 to 239.16) | 4772 (2341 to 8329) | 140.53 (68.88 to 245.35) | 0% (-0.01 to 0.01) |
| Bosnia and Herzegovina | 4185 (2239 to 6786) | 357.07 (191.21 to 579.39) | 1896 (1006 to 3093) | 355.64 (189.04 to 580.49) | -0.05% (-0.07 to -0.03) |
| Botswana | 862 (439 to 1445) | 168.14 (85.91 to 281.57) | 1212 (602 to 1999) | 174.34 (86.47 to 288.01) | 0.53% (0.3 to 0.75) |
| Brazil | 39912 (20020 to 66500) | 79.07 (39.7 to 131.82) | 60741 (33061 to 98463) | 123.6 (67.09 to 200.44) | 1.16% (0.68 to 1.64) |
| Brunei Darussalam | 99 (48 to 171) | 124.05 (59.91 to 214.35) | 127 (61 to 223) | 122.88 (58.78 to 216.21) | -0.03% (-0.06 to 0) |
| Bulgaria | 7092 (3842 to 11591) | 370.79 (200.83 to 606.56) | 3615 (1947 to 5914) | 356.81 (192.39 to 584.23) | -0.11% (-0.12 to -0.1) |
| Burkina Faso | 4587 (2283 to 7724) | 131.39 (65.95 to 220.21) | 11200 (5653 to 18974) | 133.92 (67.84 to 226.63) | 0.09% (0.08 to 0.11) |
| Burundi | 2802 (1407 to 4720) | 145.63 (73.74 to 244.35) | 7361 (3788 to 12394) | 149.84 (77.48 to 251.86) | 0.08% (0.04 to 0.11) |
| Cabo Verde | 166 (81 to 281) | 128.57 (63.31 to 217.91) | 202 (103 to 339) | 130.78 (66.44 to 220.21) | 0.1% (0.08 to 0.11) |
| Cambodia | 3359 (1574 to 5924) | 91.2 (42.96 to 160.64) | 4624 (2166 to 8200) | 94.25 (44.14 to 167.22) | 0.08% (0.06 to 0.1) |
| Cameroon | 4846 (2397 to 8175) | 130.32 (64.78 to 219.05) | 15435 (7748 to 25845) | 131.89 (66.37 to 220.62) | 0.04% (0.04 to 0.05) |
| Canada | 8179 (3941 to 13841) | 136.44 (65.46 to 231.23) | 8913 (4301 to 15078) | 135.98 (65.44 to 230.21) | 0.01% (0 to 0.02) |
| Central African Republic | 1278 (637 to 2184) | 134.6 (67.31 to 229.4) | 2830 (1419 to 4785) | 139.38 (69.98 to 235.56) | 0.11% (0.09 to 0.13) |
| Chad | 2750 (1358 to 4708) | 128.49 (63.79 to 219.64) | 9061 (4493 to 15496) | 131.58 (65.55 to 224.25) | 0.07% (0.06 to 0.08) |
| Chile | 6459 (3297 to 10951) | 161.12 (81.92 to 273.24) | 8257 (5416 to 11966) | 209.96 (137.55 to 304.38) | 0.53% (0.06 to 1.01) |
| China | 336643 (155878 to 603968) | 93.13 (42.61 to 166.97) | 136911 (65402 to 234308) | 54.68 (26.19 to 93.54) | -2.25% (-2.58 to -1.91) |
| Colombia | 12495 (6230 to 21323) | 114.85 (57.26 to 195.98) | 13251 (6601 to 22706) | 115.86 (57.77 to 198.46) | 0.05% (0.04 to 0.06) |
| Comoros | 254 (128 to 428) | 143.95 (72.77 to 242.89) | 351 (178 to 585) | 148.54 (75.5 to 247.75) | 0.08% (0.04 to 0.12) |
| Congo | 1222 (615 to 2090) | 133.29 (67.18 to 227.82) | 2501 (1263 to 4210) | 135.38 (68.45 to 227.97) | 0.08% (0.06 to 0.09) |
| Cook Islands | 6 (3 to 10) | 91.79 (42.92 to 161.72) | 4 (2 to 7) | 93.79 (44.38 to 163.12) | 0.1% (0.08 to 0.12) |
| Costa Rica | 1143 (563 to 1944) | 114.14 (56.17 to 194.07) | 1229 (611 to 2131) | 114.09 (56.7 to 197.69) | 0% (0 to 0) |
| Coted'Ivoire | 5609 (2758 to 9574) | 129.82 (64.17 to 220.63) | 12820 (6365 to 21971) | 132.67 (66.05 to 227.22) | 0.08% (0.07 to 0.09) |
| Croatia | 3789 (2048 to 6176) | 359.31 (194.27 to 585.88) | 1734 (1019 to 2698) | 267.53 (156.89 to 417.49) | -1.03% (-1.73 to -0.32) |
| Cuba | 3940 (1940 to 6741) | 128.81 (62.95 to 220.09) | 2482 (1211 to 4261) | 129.49 (63.03 to 222.44) | 0.04% (0.03 to 0.05) |
| Cyprus | 126 (54 to 226) | 63.66 (27.52 to 114.79) | 136 (59 to 247) | 63.86 (27.49 to 115.86) | -0.35% (-1.01 to 0.31) |
| Czechia | 9703 (5222 to 15827) | 382.16 (206.01 to 623.36) | 6245 (3365 to 10121) | 374.72 (202.12 to 607.83) | -0.42% (-0.68 to -0.16) |
| Democratic People's Republic of Korea | 3385 (1472 to 6007) | 59.82 (25.81 to 106.09) | 3468 (1483 to 6190) | 63.29 (26.76 to 113.01) | 0.16% (0.14 to 0.18) |
| Democratic Republic of the Congo | 18192 (9178 to 30743) | 132.6 (67.27 to 223.41) | 45546 (22805 to 77384) | 135.72 (68.1 to 230.42) | 0.1% (0.09 to 0.11) |
| Denmark | 709 (302 to 1283) | 64.83 (27.04 to 117.44) | 683 (290 to 1248) | 64.68 (27.17 to 118.17) | -0.19% (-1.24 to 0.86) |
| Djibouti | 233 (118 to 396) | 144.42 (73.01 to 245.71) | 578 (289 to 969) | 152.39 (76.26 to 255.33) | 0.17% (0.15 to 0.19) |
| Dominica | 32 (15 to 55) | 128.32 (62.33 to 221.67) | 22 (11 to 38) | 129.54 (63.65 to 221.05) | 0.03% (0.02 to 0.05) |
| Dominican Republic | 3265 (1613 to 5625) | 128.23 (63.29 to 221.01) | 3761 (1859 to 6480) | 128.28 (63.21 to 221.06) | -0.02% (-0.03 to 0) |
| Ecuador | 4304 (2301 to 7141) | 120.01 (64.22 to 199.15) | 7084 (4761 to 9882) | 141.58 (95.1 to 197.57) | 1.29% (0.97 to 1.61) |
| Egypt | 27713 (14003 to 47028) | 144.88 (73.3 to 245.79) | 47883 (24384 to 79965) | 146.49 (74.77 to 244.46) | 0.03% (0.02 to 0.04) |
| El Salvador | 2244 (1130 to 3838) | 113.18 (56.97 to 193.75) | 2034 (1003 to 3482) | 113.66 (56.06 to 194.52) | 0.02% (0.01 to 0.03) |
| Equatorial Guinea | 201 (100 to 340) | 133.39 (66.66 to 225.19) | 824 (410 to 1407) | 139.04 (69.11 to 237.76) | 0.2% (0.18 to 0.22) |
| Eritrea | 1876 (947 to 3171) | 145.38 (73.69 to 245.47) | 3425 (1735 to 5634) | 150.58 (76.37 to 247.54) | 0.08% (0.05 to 0.11) |
| Estonia | 1148 (599 to 1875) | 330.88 (172.45 to 540.75) | 685 (357 to 1141) | 322.65 (168.32 to 538.04) | -0.1% (-0.11 to -0.08) |
| Eswatini | 531 (267 to 883) | 167.35 (84.58 to 277.74) | 699 (353 to 1182) | 176.24 (88.85 to 297.94) | 0.17% (0.14 to 0.2) |
| Ethiopia | 34548 (17755 to 56859) | 186.16 (96.21 to 305.46) | 72134 (37427 to 119667) | 173.88 (90.18 to 288.54) | -0.27% (-0.31 to -0.23) |
| Fiji | 229 (106 to 395) | 88.09 (40.86 to 151.66) | 240 (113 to 416) | 92.77 (43.69 to 161.03) | 0.14% (0.13 to 0.15) |
| Finland | 622 (267 to 1121) | 63.77 (27.29 to 114.89) | 610 (262 to 1092) | 64.15 (27.28 to 114.87) | 0.61% (-0.6 to 1.84) |
| France | 9638 (4186 to 17246) | 72.06 (30.87 to 128.88) | 9326 (4051 to 16645) | 71.99 (31.02 to 128.54) | 0% (-0.01 to 0) |
| Gabon | 452 (228 to 778) | 132.17 (66.74 to 227.14) | 824 (411 to 1393) | 134.29 (67.02 to 227.02) | 0.04% (0.03 to 0.05) |
| Gambia | 459 (229 to 781) | 129.27 (64.69 to 219.47) | 1180 (587 to 2003) | 130.86 (65.19 to 222.03) | 0.06% (0.05 to 0.06) |
| Georgia | 4335 (2292 to 7213) | 320.73 (169.54 to 533.47) | 960 (627 to 1401) | 144.71 (94.75 to 210.81) | -3.4% (-3.97 to -2.83) |
| Germany | 8949 (3811 to 16215) | 65.8 (27.74 to 119.3) | 8198 (3566 to 14647) | 65.79 (28.39 to 117.67) | 0.57% (0.17 to 0.97) |
| Ghana | 7108 (3547 to 12064) | 132.46 (66.42 to 224.48) | 16127 (8187 to 26983) | 140.89 (71.66 to 235.49) | 0.21% (0.17 to 0.26) |
| Greece | 1899 (847 to 3383) | 78.11 (34.47 to 139.21) | 1865 (1011 to 2976) | 117.42 (63.43 to 187.5) | 1.99% (1.71 to 2.26) |
| Greenland | 16 (8 to 28) | 134.89 (66.4 to 232.43) | 16 (7 to 26) | 134.36 (64.51 to 228.88) | -0.02% (-0.03 to -0.01) |
| Grenada | 38 (19 to 64) | 128.21 (63.01 to 217.85) | 32 (16 to 55) | 131.19 (65.3 to 223.33) | 0.08% (0.07 to 0.09) |
| Guam | 35 (16 to 60) | 89.39 (41.26 to 156.25) | 34 (16 to 59) | 92.47 (43.35 to 160.99) | 0.11% (0.1 to 0.12) |
| Guatemala | 3845 (1929 to 6564) | 117.45 (58.9 to 200.91) | 5934 (2958 to 10206) | 115.83 (57.72 to 198.99) | -0.04% (-0.06 to -0.02) |
| Guinea | 2550 (1249 to 4339) | 129.73 (63.97 to 220.16) | 6540 (3256 to 11114) | 131.44 (65.8 to 223.26) | 0.04% (0.03 to 0.06) |
| Guinea-Bissau | 494 (247 to 833) | 131 (65.83 to 220.54) | 995 (490 to 1687) | 131.21 (64.81 to 222.11) | 0.02% (0 to 0.03) |
| Guyana | 353 (173 to 605) | 128.29 (62.73 to 219.92) | 277 (135 to 468) | 131.76 (64.11 to 222.89) | 0.09% (0.08 to 0.1) |
| Haiti | 2950 (1451 to 5133) | 133.33 (65.73 to 232) | 5465 (2702 to 9395) | 135.01 (66.75 to 232.08) | 0.07% (0.04 to 0.09) |
| Honduras | 2162 (1074 to 3659) | 117.15 (58.24 to 198.27) | 3818 (1903 to 6503) | 115.7 (57.63 to 196.91) | -0.08% (-0.09 to -0.06) |
| Hungary | 8909 (4796 to 14539) | 371.35 (200.31 to 606.93) | 5381 (2944 to 8720) | 364.03 (199.11 to 590.72) | -0.08% (-0.09 to -0.07) |
| Iceland | 43 (19 to 77) | 65.11 (28.48 to 116.92) | 45 (18 to 81) | 65.12 (26.48 to 117.65) | 0.25% (0.07 to 0.44) |
| India | 689911 (358348 to 1142451) | 242.52 (126.11 to 401.21) | 1064248 (554992 to 1740564) | 258.25 (134.43 to 422.91) | 0.32% (0.25 to 0.39) |
| Indonesia | 78894 (37193 to 137326) | 121.45 (57.28 to 211.49) | 47367 (23018 to 79319) | 67.12 (32.49 to 112.43) | -2.66% (-3.11 to -2.2) |
| Iran (Islamic Republic of) | 36498 (18598 to 60627) | 169.6 (86.69 to 280.85) | 33748 (17381 to 56121) | 175.34 (90.48 to 291.41) | 0.11% (0.1 to 0.13) |
| Iraq | 10282 (5200 to 17357) | 147.63 (74.81 to 248.85) | 19677 (9889 to 33251) | 146.13 (73.42 to 247.09) | -0.05% (-0.07 to -0.04) |
| Ireland | 788 (344 to 1412) | 72.23 (31.26 to 129.52) | 756 (334 to 1341) | 72.22 (31.87 to 128.09) | -0.03% (-0.04 to -0.02) |
| Israel | 1099 (474 to 1952) | 72.43 (31.13 to 128.62) | 1740 (758 to 3122) | 71.41 (31.14 to 128.14) | -0.02% (-0.03 to -0.01) |
| Italy | 14861 (7121 to 25671) | 118.97 (56.41 to 206.13) | 9106 (5633 to 13543) | 103.28 (63.77 to 153.71) | -0.8% (-1.02 to -0.57) |
| Jamaica | 1050 (510 to 1798) | 127.66 (62 to 218.76) | 891 (437 to 1528) | 130.21 (63.7 to 223.37) | 0.08% (0.07 to 0.09) |
| Japan | 46303 (23131 to 78391) | 159.06 (78.98 to 269.51) | 21007 (11105 to 34250) | 120.21 (63.26 to 196.24) | -1.41% (-1.71 to -1.11) |
| Jordan | 2196 (1099 to 3706) | 146.88 (73.47 to 247.81) | 6919 (3648 to 11458) | 173.3 (91.18 to 287.32) | 0.78% (0.63 to 0.92) |
| Kazakhstan | 16774 (8749 to 27674) | 353.71 (184.5 to 583.61) | 16294 (8659 to 26882) | 355.36 (189 to 587.1) | 0.03% (0.02 to 0.04) |
| Kenya | 15097 (7727 to 25128) | 165.34 (84.95 to 274.88) | 32489 (16701 to 53426) | 171.05 (87.85 to 281.63) | 0.1% (0.08 to 0.12) |
| Kiribati | 22 (10 to 38) | 89.72 (41.95 to 157.26) | 36 (17 to 62) | 93.5 (43.5 to 161.94) | 0.13% (0.12 to 0.15) |
| Kuwait | 658 (331 to 1121) | 142.65 (71.88 to 242.8) | 1214 (612 to 2043) | 146.6 (74.01 to 246.95) | 0.04% (0.01 to 0.06) |
| Kyrgyzstan | 5850 (3148 to 9493) | 405.22 (217.97 to 657.37) | 7743 (4121 to 12829) | 399.03 (212.46 to 661.52) | 0.27% (0.05 to 0.49) |
| Lao People's Democratic Republic | 1416 (665 to 2478) | 94.45 (44.51 to 165.28) | 2062 (967 to 3626) | 94.05 (44.04 to 165.38) | -0.04% (-0.07 to -0.01) |
| Latvia | 1822 (942 to 3039) | 321.73 (166.31 to 536.18) | 922 (482 to 1512) | 313.65 (164.17 to 514.48) | -0.56% (-0.89 to -0.22) |
| Lebanon | 1369 (682 to 2310) | 144.2 (71.81 to 243.24) | 1860 (921 to 3179) | 147.01 (72.83 to 251.43) | 0.07% (0.05 to 0.08) |
| Lesotho | 933 (466 to 1533) | 167.05 (83.76 to 274.06) | 1153 (587 to 1919) | 176.31 (89.5 to 293.95) | 0.18% (0.16 to 0.2) |
| Liberia | 1090 (532 to 1865) | 129.43 (63.42 to 221.06) | 2634 (1313 to 4455) | 131.17 (65.46 to 221.9) | 0.08% (0.07 to 0.1) |
| Libya | 2357 (1174 to 3975) | 142.08 (70.86 to 239.78) | 2574 (1290 to 4279) | 144.9 (72.34 to 241.4) | 0.07% (0.05 to 0.1) |
| Lithuania | 3038 (1601 to 4985) | 356.99 (188.02 to 585.96) | 1380 (715 to 2319) | 337.1 (174.61 to 566.34) | -0.45% (-0.79 to -0.12) |
| Luxembourg | 56 (25 to 101) | 81.1 (35.87 to 145.91) | 86 (38 to 152) | 80.9 (35.92 to 143.52) | -0.36% (-0.72 to 0.01) |
| Madagascar | 6479 (3199 to 10847) | 147.94 (73.38 to 247.23) | 15941 (8023 to 26895) | 149.11 (75.17 to 251.42) | 0.03% (-0.02 to 0.07) |
| Malawi | 5160 (2595 to 8754) | 146.57 (74.02 to 248.12) | 11966 (6101 to 20098) | 150.1 (76.48 to 252.37) | 0.07% (0.03 to 0.1) |
| Malaysia | 4974 (2310 to 8816) | 84.57 (39.32 to 149.86) | 7134 (3252 to 12601) | 88.33 (40.17 to 155.91) | 0.14% (0.13 to 0.15) |
| Maldives | 69 (32 to 120) | 84.22 (39.42 to 145.81) | 87 (41 to 151) | 88.66 (41.14 to 153.49) | 0.21% (0.19 to 0.22) |
| Mali | 3906 (1917 to 6696) | 130.92 (64.63 to 224.06) | 12181 (6029 to 20561) | 132.05 (65.55 to 222.47) | 0.05% (0.03 to 0.06) |
| Malta | 69 (31 to 123) | 77.4 (34.26 to 138.28) | 50 (22 to 88) | 77.97 (34.16 to 138.9) | 0.62% (-0.09 to 1.33) |
| Marshall Islands | 16 (7 to 28) | 89.44 (41.47 to 153.54) | 17 (8 to 29) | 94.47 (44.31 to 164.27) | 0.16% (0.15 to 0.17) |
| Mauritania | 943 (469 to 1578) | 129.56 (64.65 to 216.32) | 2144 (1067 to 3656) | 131.45 (65.58 to 224) | 0.03% (0.02 to 0.04) |
| Mauritius | 285 (130 to 497) | 86.39 (39.5 to 150.93) | 224 (103 to 394) | 88.59 (40.5 to 156.2) | 0.08% (0.06 to 0.09) |
| Mexico | 51346 (25884 to 87631) | 161.51 (81.43 to 275.73) | 51367 (28374 to 83674) | 150.79 (83.23 to 245.68) | -0.48% (-1.16 to 0.2) |
| Micronesia (Federated States of) | 37 (17 to 65) | 90.27 (42.03 to 159.23) | 31 (14 to 55) | 94.51 (43.41 to 164.66) | 0.13% (0.12 to 0.14) |
| Monaco | 3 (1 to 5) | 72.18 (31.86 to 127.19) | 4 (2 to 7) | 71.67 (31.21 to 128.35) | -0.01% (-0.02 to 0) |
| Mongolia | 2801 (1496 to 4630) | 357 (190.61 to 589.87) | 3022 (1609 to 5018) | 355.62 (189.09 to 589.43) | -0.02% (-0.03 to -0.01) |
| Montenegro | 595 (321 to 973) | 359.44 (193.85 to 587.88) | 422 (228 to 684) | 356.04 (192.13 to 576.92) | -0.05% (-0.06 to -0.03) |
| Morocco | 12560 (6187 to 21159) | 141.67 (69.83 to 238.73) | 14324 (7208 to 23968) | 145.21 (72.96 to 243.18) | 0.1% (0.09 to 0.11) |
| Mozambique | 7061 (3530 to 11927) | 145.13 (72.96 to 244.74) | 18239 (9337 to 30532) | 151.51 (77.93 to 253.06) | 0.14% (0.1 to 0.17) |
| Myanmar | 13174 (6149 to 23163) | 93.41 (43.6 to 164.28) | 15076 (7060 to 26499) | 94.31 (44.06 to 165.87) | 0.02% (-0.02 to 0.05) |
| Namibia | 891 (448 to 1494) | 168.03 (84.58 to 281.57) | 1371 (694 to 2282) | 171.69 (86.85 to 285.84) | 0.07% (0.07 to 0.08) |
| Nauru | 3 (1 to 5) | 89.67 (41.57 to 155.1) | 4 (2 to 6) | 95.18 (44.76 to 165.81) | 0.17% (0.16 to 0.18) |
| Nepal | 13266 (6722 to 21922) | 198.22 (100.75 to 327.14) | 19592 (10068 to 32494) | 198.48 (101.65 to 329.59) | 0.04% (0.02 to 0.05) |
| Netherlands | 2396 (1047 to 4254) | 73.18 (31.4 to 129.88) | 2255 (1014 to 4005) | 73.12 (32.48 to 129.99) | -0.01% (-0.02 to 0) |
| New Zealand | 817 (364 to 1478) | 91.53 (40.32 to 165.63) | 714 (383 to 1171) | 69.6 (37.23 to 114.19) | -0.89% (-1.23 to -0.54) |
| Nicaragua | 1763 (878 to 3025) | 113.58 (56.6 to 195.21) | 2241 (1120 to 3830) | 114.25 (57.08 to 195.31) | 0.02% (0.02 to 0.03) |
| Niger | 3777 (1861 to 6471) | 129.67 (64.34 to 221.3) | 13000 (6405 to 22232) | 130.81 (64.77 to 223.18) | 0.04% (0.03 to 0.06) |
| Nigeria | 47655 (24090 to 80280) | 149.75 (75.84 to 252.12) | 133217 (67629 to 222002) | 149.99 (76.32 to 249.92) | -0.01% (-0.03 to 0.02) |
| Niue | 1 (0 to 1) | 90.23 (41.5 to 158.85) | 0 (0 to 1) | 94.8 (44.42 to 164.27) | 0.15% (0.12 to 0.17) |
| North Macedonia | 1934 (1043 to 3150) | 360.59 (194.42 to 587.44) | 1278 (688 to 2068) | 355.53 (191.32 to 575.43) | -0.06% (-0.07 to -0.04) |
| Northern Mariana Islands | 10 (5 to 18) | 87.25 (40.04 to 152.06) | 11 (5 to 19) | 92.74 (42.76 to 160.13) | 0.21% (0.16 to 0.26) |
| Norway | 1486 (733 to 2530) | 161.5 (79.16 to 275.31) | 1671 (820 to 2845) | 167.47 (82.12 to 285.4) | 0.8% (0.31 to 1.3) |
| Oman | 907 (450 to 1525) | 146.76 (73.34 to 246.61) | 1449 (724 to 2450) | 146.38 (73.52 to 247.05) | 0.06% (0.04 to 0.08) |
| Pakistan | 95750 (49220 to 158366) | 235.22 (121.08 to 388.58) | 192433 (101241 to 314293) | 244.52 (128.71 to 399.32) | 0.12% (0.11 to 0.13) |
| Palau | 4 (2 to 7) | 89.26 (41.97 to 153.23) | 3 (2 to 6) | 93.65 (44.01 to 161.63) | 0.17% (0.16 to 0.18) |
| Palestine | 1128 (563 to 1910) | 145.84 (73.05 to 246.56) | 2613 (1317 to 4389) | 146.24 (73.79 to 245.74) | -0.01% (-0.02 to 0) |
| Panama | 924 (459 to 1588) | 114.71 (56.93 to 197.1) | 1296 (654 to 2214) | 113.77 (57.38 to 194.41) | -0.02% (-0.02 to -0.01) |
| Papua New Guinea | 1323 (607 to 2303) | 91.24 (41.9 to 158.8) | 3174 (1503 to 5431) | 95.92 (45.51 to 164.01) | 0.17% (0.16 to 0.19) |
| Paraguay | 1473 (702 to 2555) | 106.76 (51.14 to 185.04) | 2203 (1048 to 3770) | 105.61 (50.05 to 180.89) | -0.03% (-0.04 to -0.03) |
| Peru | 10749 (5228 to 18531) | 139.66 (67.96 to 240.81) | 13259 (6487 to 22708) | 140.93 (68.82 to 241.35) | 0.02% (0.01 to 0.03) |
| Philippines | 29417 (14264 to 50454) | 130.11 (63.13 to 223.16) | 55779 (28096 to 93557) | 162.76 (81.87 to 273.23) | 1.01% (0.82 to 1.21) |
| Poland | 36269 (19075 to 59895) | 381.98 (200.96 to 631.09) | 15694 (10233 to 22730) | 266.82 (174.08 to 386.59) | -1.5% (-1.85 to -1.15) |
| Portugal | 1708 (722 to 3081) | 64.51 (26.84 to 116.39) | 1243 (627 to 2050) | 74.43 (36.94 to 123.04) | 0.67% (0.55 to 0.8) |
| Puerto Rico | 1342 (653 to 2286) | 128.22 (62.19 to 218.65) | 772 (385 to 1332) | 129.16 (64 to 222.78) | 0.04% (0.03 to 0.05) |
| Qatar | 144 (72 to 242) | 147.08 (73.85 to 247.49) | 558 (277 to 938) | 145.16 (72.62 to 243.53) | 0.07% (0.02 to 0.13) |
| Republic of Korea | 16329 (7865 to 28108) | 121.73 (58.34 to 209.93) | 8474 (4077 to 14521) | 119.22 (57.18 to 204.64) | -0.05% (-0.05 to -0.04) |
| Republic of Moldova | 3784 (1972 to 6322) | 332.72 (173.34 to 555.78) | 1795 (932 to 2986) | 323.06 (167.74 to 537.81) | -0.11% (-0.13 to -0.08) |
| Romania | 21796 (11694 to 35259) | 364.36 (195.89 to 590.03) | 11600 (6293 to 18657) | 361.49 (196.33 to 581.93) | 0.04% (-0.01 to 0.09) |
| Russian Federation | 138060 (73792 to 225966) | 409.45 (218.8 to 670.08) | 102097 (54746 to 166510) | 396.07 (212.5 to 645.9) | -0.14% (-0.15 to -0.12) |
| Rwanda | 3853 (1928 to 6480) | 146.9 (73.93 to 246.27) | 7011 (3551 to 11662) | 148.38 (75.1 to 246.92) | 0.03% (0 to 0.06) |
| Saint Kitts and Nevis | 18 (9 to 30) | 127.41 (62.99 to 219.1) | 15 (8 to 27) | 129.36 (62.92 to 224.28) | 0.05% (0.04 to 0.07) |
| Saint Lucia | 63 (31 to 108) | 127.93 (63.13 to 219.77) | 46 (23 to 79) | 130.56 (65.05 to 224.35) | 0.1% (0.09 to 0.11) |
| Saint Vincent and the Grenadines | 53 (26 to 91) | 128.49 (63.03 to 219.99) | 35 (18 to 60) | 129.57 (64.48 to 219.31) | 0.05% (0.04 to 0.06) |
| Samoa | 60 (28 to 104) | 90.57 (42.06 to 156.14) | 66 (31 to 114) | 93.34 (43.37 to 161.58) | 0.12% (0.1 to 0.13) |
| San Marino | 4 (2 to 7) | 74.06 (32.04 to 131.26) | 5 (2 to 8) | 80.17 (35.63 to 143.9) | 0.27% (0.25 to 0.3) |
| Sao Tome and Principe | 61 (31 to 103) | 129.44 (65.55 to 219.62) | 103 (50 to 174) | 131.88 (64.83 to 224.08) | 0.06% (0.05 to 0.07) |
| Saudi Arabia | 8132 (4097 to 13623) | 144.93 (73.14 to 242.49) | 11816 (5950 to 20057) | 148.87 (74.74 to 252.78) | 0.11% (0.09 to 0.13) |
| Senegal | 3648 (1786 to 6220) | 129.53 (63.72 to 220.53) | 7705 (3831 to 13193) | 132.83 (66.11 to 227.49) | 0.11% (0.1 to 0.13) |
| Serbia | 8336 (4465 to 13572) | 364.81 (195.63 to 594.13) | 5787 (3079 to 9492) | 358.74 (191.1 to 588.93) | -0.4% (-0.65 to -0.15) |
| Seychelles | 20 (9 to 36) | 85.46 (39.81 to 152.76) | 21 (10 to 36) | 89.98 (42.05 to 157.68) | 0.17% (0.16 to 0.17) |
| Sierra Leone | 1764 (864 to 2990) | 129.66 (63.69 to 219.13) | 4161 (2090 to 7063) | 130.74 (65.69 to 221.77) | 0.06% (0.05 to 0.07) |
| Singapore | 933 (456 to 1612) | 120.23 (58.24 to 207.52) | 894 (430 to 1549) | 119.68 (57.57 to 206.82) | -0.03% (-0.04 to -0.01) |
| Slovakia | 5695 (3070 to 9291) | 414.93 (223.87 to 677.77) | 3470 (1876 to 5625) | 409.03 (221.41 to 663.56) | -0.12% (-0.61 to 0.37) |
| Slovenia | 1518 (816 to 2483) | 339.45 (182.71 to 555.39) | 1035 (551 to 1710) | 337.46 (179.88 to 557.65) | -0.89% (-1.91 to 0.13) |
| Solomon Islands | 120 (56 to 207) | 90.49 (42.28 to 156.68) | 224 (106 to 387) | 95.81 (45.6 to 165.89) | 0.18% (0.18 to 0.19) |
| Somalia | 4491 (2242 to 7557) | 149.3 (74.95 to 250.49) | 13158 (6766 to 21981) | 158.22 (81.61 to 263.51) | 0.17% (0.13 to 0.21) |
| South Africa | 24728 (12775 to 40660) | 192.68 (99.48 to 316.87) | 29084 (15038 to 48100) | 192.12 (99.29 to 317.94) | 0.01% (-0.01 to 0.03) |
| South Sudan | 3222 (1627 to 5401) | 145.14 (73.43 to 243.14) | 5833 (2955 to 9789) | 148.44 (75.23 to 249.34) | 0.05% (0.03 to 0.08) |
| Spain | 6561 (2805 to 11705) | 64.99 (27.29 to 116.04) | 7912 (4066 to 12537) | 107.99 (55.2 to 171.38) | 2.37% (1.91 to 2.83) |
| Sri Lanka | 4655 (2119 to 8154) | 84.41 (38.42 to 147.92) | 4884 (2264 to 8486) | 88.27 (40.82 to 153.4) | 0.14% (0.13 to 0.15) |
| Sudan | 10495 (5258 to 17612) | 142.82 (71.74 to 239.51) | 23018 (11491 to 38623) | 146.46 (73.15 to 245.85) | 0.1% (0.09 to 0.12) |
| Suriname | 166 (81 to 284) | 129.02 (63.19 to 221.33) | 195 (96 to 336) | 131.9 (64.87 to 227.08) | 0.08% (0.08 to 0.09) |
| Sweden | 1550 (719 to 2697) | 92.69 (42.61 to 161.36) | 2376 (1145 to 4094) | 126.4 (60.81 to 217.92) | 0.78% (0.59 to 0.97) |
| Switzerland | 990 (448 to 1741) | 77.35 (34.54 to 136.06) | 1049 (465 to 1892) | 76.91 (33.91 to 138.76) | -0.72% (-1.1 to -0.35) |
| Syrian Arab Republic | 7155 (3576 to 12167) | 143.33 (71.94 to 243.59) | 7179 (3658 to 11967) | 143.81 (72.67 to 240.58) | -0.03% (-0.06 to 0) |
| Taiwan (Province of China) | 3693 (1623 to 6554) | 63.6 (27.9 to 112.96) | 2668 (1438 to 4265) | 80.2 (42.95 to 128.16) | 0.86% (0.68 to 1.05) |
| Tajikistan | 6692 (3510 to 11204) | 358.27 (187.72 to 599.55) | 10991 (5796 to 18240) | 365.99 (192.86 to 607.41) | 0.06% (0.05 to 0.07) |
| Thailand | 15612 (7249 to 27444) | 85.78 (39.71 to 150.93) | 10106 (4764 to 17618) | 87.93 (41.23 to 153.4) | 0.08% (0.07 to 0.09) |
| Timor-Leste | 230 (104 to 405) | 90.43 (40.94 to 158.5) | 475 (221 to 828) | 92.54 (43.07 to 161.45) | 0.06% (0.02 to 0.1) |
| Togo | 1791 (878 to 3040) | 129.43 (63.76 to 219.36) | 3883 (1897 to 6644) | 132.5 (64.86 to 226.65) | 0.1% (0.08 to 0.12) |
| Tokelau | 0 (0 to 1) | 89.17 (41.77 to 156.53) | 0 (0 to 1) | 93.93 (43.35 to 166.04) | 0.16% (0.14 to 0.18) |
| Tonga | 34 (16 to 59) | 90.36 (42.26 to 156.4) | 33 (15 to 58) | 94.78 (43.79 to 166.08) | 0.14% (0.14 to 0.15) |
| Trinidad and Tobago | 486 (236 to 831) | 128.45 (62.39 to 219.27) | 374 (185 to 640) | 129.23 (63.94 to 221.44) | 0.04% (0.03 to 0.04) |
| Tunisia | 4177 (2083 to 7051) | 143.62 (71.67 to 242.47) | 3911 (1978 to 6642) | 145.54 (73.61 to 247.22) | 0.05% (0.04 to 0.06) |
| Turkey | 29016 (14628 to 48576) | 145.24 (73.19 to 243.37) | 28639 (14427 to 48061) | 146.35 (73.63 to 245.78) | 0% (0 to 0.01) |
| Turkmenistan | 4566 (2421 to 7653) | 357.7 (189.65 to 599.3) | 5156 (2780 to 8577) | 363.66 (196.04 to 604.81) | 0.07% (0.06 to 0.07) |
| Tuvalu | 2 (1 to 4) | 90.37 (41.59 to 158.13) | 3 (2 to 6) | 94.08 (44.19 to 164.94) | 0.11% (0.1 to 0.13) |
| Uganda | 9205 (4559 to 15404) | 143 (71.07 to 238.73) | 25507 (12751 to 42245) | 148.25 (74.29 to 245.31) | 0.1% (0.07 to 0.12) |
| Ukraine | 44997 (24108 to 74546) | 389.48 (208.52 to 645) | 26156 (13777 to 43200) | 377.5 (199.14 to 624.43) | -0.13% (-0.14 to -0.12) |
| United Arab Emirates | 632 (318 to 1071) | 145.48 (73.78 to 245.54) | 1783 (890 to 2999) | 146.3 (73.21 to 245.9) | 0.02% (-0.05 to 0.08) |
| United Kingdom | 12367 (5729 to 21799) | 105.14 (48.42 to 185.32) | 10779 (5352 to 18316) | 86.8 (42.98 to 147.57) | 0.23% (-0.18 to 0.63) |
| United Republic of Tanzania | 13934 (6985 to 23709) | 142.75 (71.82 to 242.69) | 31512 (15853 to 53011) | 145.94 (73.57 to 245.39) | 0.07% (0.05 to 0.1) |
| United States of America | 87290 (42909 to 148921) | 153.51 (75.18 to 262.16) | 87559 (50809 to 135818) | 129.67 (74.92 to 201.23) | -1.44% (-1.97 to -0.9) |
| United States Virgin Islands | 41 (20 to 69) | 130.43 (63.78 to 221.29) | 20 (10 to 34) | 132.02 (64.95 to 225.5) | 0.05% (0.04 to 0.06) |
| Uruguay | 1341 (663 to 2267) | 162.45 (80.2 to 274.86) | 1229 (614 to 2082) | 164.4 (81.77 to 278.63) | 0.02% (0.01 to 0.04) |
| Uzbekistan | 25067 (13443 to 41788) | 352.16 (188.81 to 586.91) | 30292 (15992 to 50080) | 355.09 (187.42 to 586.96) | 0.05% (0.03 to 0.06) |
| Vanuatu | 48 (22 to 83) | 88.93 (40.96 to 154.26) | 98 (46 to 168) | 94.48 (44.83 to 162.8) | 0.17% (0.16 to 0.18) |
| Venezuela (Bolivarian Republic of) | 7726 (3861 to 13244) | 118.25 (59.09 to 202.77) | 8138 (4064 to 13658) | 122.39 (61.08 to 205.4) | 0.12% (0.08 to 0.16) |
| Viet Nam | 22939 (10681 to 40015) | 96.12 (44.79 to 167.69) | 29966 (15323 to 49033) | 129.93 (66.55 to 212.49) | 1.35% (1.13 to 1.57) |
| Yemen | 7414 (3687 to 12579) | 144.57 (72.27 to 245.35) | 18313 (9106 to 31185) | 145.97 (72.74 to 248.68) | 0% (-0.01 to 0.01) |
| Zambia | 4575 (2317 to 7641) | 145.41 (73.77 to 242.71) | 11060 (5591 to 18462) | 150.42 (76.18 to 250.67) | 0.09% (0.06 to 0.11) |
| Zimbabwe | 7374 (3785 to 12221) | 179.24 (92.32 to 296.48) | 11570 (5983 to 19228) | 204.68 (106.07 to 340.03) | 0.43% (0.37 to 0.48) |

***Table S2. pediatric stone disease Deaths for both sexes and for 204 countries in 1990 and 2021, and estimated annual percentage changes from 1990 to 2021.***

| location | Num_1990 | ASR_1990 | Num_2021 | ASR_2021 | EAPC_CI |
| --- | --- | --- | --- | --- | --- |
| Afghanistan | 0 (0 to 0) | 0 (0 to 0.01) | 1 (0 to 2) | 0.01 (0 to 0.02) | 4.49% (3.6 to 5.37) |
| Albania | 0 (0 to 0) | 0 (0 to 0.01) | 0 (0 to 0) | 0 (0 to 0) | -5.79% (-6.13 to -5.44) |
| Algeria | 0 (0 to 0) | 0 (0 to 0) | 0 (0 to 1) | 0 (0 to 0.01) | 2.59% (1.89 to 3.31) |
| American Samoa | 0 (0 to 0) | 0 (0 to 0) | 0 (0 to 0) | 0 (0 to 0) | -1.69% (-2.4 to -0.99) |
| Andorra | 0 (0 to 0) | 0 (0 to 0) | 0 (0 to 0) | 0 (0 to 0) | -2.28% (-2.58 to -1.97) |
| Angola | 0 (0 to 0) | 0 (0 to 0.01) | 0 (0 to 0) | 0 (0 to 0) | -1.84% (-1.97 to -1.7) |
| Antigua and Barbuda | 0 (0 to 0) | 0 (0 to 0) | 0 (0 to 0) | 0 (0 to 0) | 2.97% (1.91 to 4.04) |
| Argentina | 0 (0 to 0) | 0 (0 to 0) | 0 (0 to 0) | 0 (0 to 0) | 1.58% (0.8 to 2.36) |
| Armenia | 0 (0 to 0) | 0.01 (0.01 to 0.02) | 0 (0 to 0) | 0.01 (0 to 0.01) | -1.42% (-2.25 to -0.58) |
| Australia | 0 (0 to 0) | 0 (0 to 0) | 0 (0 to 0) | 0 (0 to 0) | -2.06% (-2.8 to -1.31) |
| Austria | 0 (0 to 0) | 0 (0 to 0) | 0 (0 to 0) | 0 (0 to 0) | -0.79% (-1.01 to -0.57) |
| Azerbaijan | 0 (0 to 0) | 0 (0 to 0) | 0 (0 to 0) | 0 (0 to 0) | -1.58% (-2.03 to -1.13) |
| Bahamas | 0 (0 to 0) | 0 (0 to 0) | 0 (0 to 0) | 0 (0 to 0) | 2.5% (1.52 to 3.49) |
| Bahrain | 0 (0 to 0) | 0 (0 to 0) | 0 (0 to 0) | 0 (0 to 0) | 4.19% (3.17 to 5.21) |
| Bangladesh | 3 (0 to 7) | 0.01 (0 to 0.02) | 1 (0 to 3) | 0 (0 to 0.01) | -2.19% (-2.44 to -1.93) |
| Barbados | 0 (0 to 0) | 0 (0 to 0) | 0 (0 to 0) | 0 (0 to 0) | 1.83% (0.99 to 2.68) |
| Belarus | 0 (0 to 0) | 0 (0 to 0.01) | 0 (0 to 0) | 0 (0 to 0) | -2.05% (-2.29 to -1.82) |
| Belgium | 0 (0 to 0) | 0 (0 to 0) | 0 (0 to 0) | 0 (0 to 0) | -1.99% (-2.27 to -1.7) |
| Belize | 0 (0 to 0) | 0 (0 to 0) | 0 (0 to 0) | 0 (0 to 0) | 4.04% (3.21 to 4.89) |
| Benin | 0 (0 to 0) | 0.01 (0 to 0.01) | 0 (0 to 0) | 0 (0 to 0.01) | -1.27% (-1.38 to -1.17) |
| Bermuda | 0 (0 to 0) | 0 (0 to 0) | 0 (0 to 0) | 0 (0 to 0) | 4.27% (2.89 to 5.67) |
| Bhutan | 0 (0 to 0) | 0 (0 to 0.01) | 0 (0 to 0) | 0 (0 to 0.01) | -2.07% (-2.29 to -1.85) |
| Bolivia (Plurinational State of) | 0 (0 to 0) | 0 (0 to 0) | 0 (0 to 0) | 0 (0 to 0) | -1.75% (-1.83 to -1.67) |
| Bosnia and Herzegovina | 0 (0 to 0) | 0 (0 to 0.01) | 0 (0 to 0) | 0 (0 to 0) | -3.88% (-4.49 to -3.28) |
| Botswana | 0 (0 to 0) | 0 (0 to 0.01) | 0 (0 to 0) | 0 (0 to 0.01) | -0.48% (-0.8 to -0.16) |
| Brazil | 2 (1 to 2) | 0 (0 to 0) | 4 (4 to 5) | 0.01 (0.01 to 0.01) | 4.12% (3.8 to 4.45) |
| Brunei Darussalam | 0 (0 to 0) | 0 (0 to 0.01) | 0 (0 to 0) | 0 (0 to 0) | -2.67% (-3.04 to -2.3) |
| Bulgaria | 0 (0 to 0) | 0.02 (0.01 to 0.02) | 0 (0 to 0) | 0 (0 to 0) | -6.32% (-7.16 to -5.47) |
| Burkina Faso | 0 (0 to 0) | 0 (0 to 0.01) | 0 (0 to 1) | 0 (0 to 0.01) | -0.71% (-0.87 to -0.54) |
| Burundi | 0 (0 to 1) | 0.01 (0 to 0.03) | 0 (0 to 1) | 0.01 (0 to 0.01) | -2.21% (-2.32 to -2.09) |
| Cabo Verde | 0 (0 to 0) | 0 (0 to 0) | 0 (0 to 0) | 0 (0 to 0) | -0.32% (-0.83 to 0.19) |
| Cambodia | 0 (0 to 1) | 0.01 (0 to 0.02) | 0 (0 to 1) | 0 (0 to 0.01) | -2.64% (-2.84 to -2.44) |
| Cameroon | 0 (0 to 0) | 0.01 (0 to 0.01) | 1 (0 to 1) | 0 (0 to 0.01) | -1.13% (-1.34 to -0.92) |
| Canada | 0 (0 to 0) | 0 (0 to 0) | 0 (0 to 0) | 0 (0 to 0) | 0.94% (0.53 to 1.35) |
| Central African Republic | 0 (0 to 0) | 0 (0 to 0.01) | 0 (0 to 0) | 0 (0 to 0) | -0.65% (-0.77 to -0.54) |
| Chad | 0 (0 to 0) | 0 (0 to 0.01) | 0 (0 to 1) | 0 (0 to 0.01) | 0.08% (-0.13 to 0.28) |
| Chile | 0 (0 to 0) | 0 (0 to 0) | 0 (0 to 0) | 0 (0 to 0) | 1.35% (0.45 to 2.24) |
| China | 44 (13 to 64) | 0.01 (0 to 0.02) | 4 (2 to 7) | 0 (0 to 0) | -7.08% (-7.34 to -6.82) |
| Colombia | 0 (0 to 0) | 0 (0 to 0) | 0 (0 to 0) | 0 (0 to 0) | -2.04% (-2.28 to -1.8) |
| Comoros | 0 (0 to 0) | 0.01 (0 to 0.02) | 0 (0 to 0) | 0.01 (0 to 0.01) | -1.4% (-2.24 to -0.55) |
| Congo | 0 (0 to 0) | 0 (0 to 0) | 0 (0 to 0) | 0 (0 to 0) | -1.27% (-1.52 to -1.03) |
| Cook Islands | 0 (0 to 0) | 0 (0 to 0) | 0 (0 to 0) | 0 (0 to 0) | -4.3% (-4.59 to -4.01) |
| Costa Rica | 0 (0 to 0) | 0 (0 to 0) | 0 (0 to 0) | 0 (0 to 0) | -0.83% (-1.13 to -0.53) |
| Coted'Ivoire | 0 (0 to 0) | 0 (0 to 0.01) | 0 (0 to 1) | 0 (0 to 0.01) | -0.68% (-0.89 to -0.47) |
| Croatia | 0 (0 to 0) | 0 (0 to 0) | 0 (0 to 0) | 0 (0 to 0) | -7.52% (-8.48 to -6.56) |
| Cuba | 0 (0 to 0) | 0 (0 to 0) | 0 (0 to 0) | 0 (0 to 0) | 1.4% (1 to 1.79) |
| Cyprus | 0 (0 to 0) | 0 (0 to 0) | 0 (0 to 0) | 0 (0 to 0) | -3.13% (-4.03 to -2.22) |
| Czechia | 0 (0 to 0) | 0.01 (0.01 to 0.01) | 0 (0 to 0) | 0 (0 to 0) | -7.72% (-8.52 to -6.92) |
| Democratic People's Republic of Korea | 0 (0 to 1) | 0.01 (0 to 0.02) | 0 (0 to 0) | 0 (0 to 0.01) | -2.75% (-2.97 to -2.54) |
| Democratic Republic of the Congo | 0 (0 to 1) | 0 (0 to 0) | 0 (0 to 1) | 0 (0 to 0) | -1.02% (-1.15 to -0.89) |
| Denmark | 0 (0 to 0) | 0 (0 to 0) | 0 (0 to 0) | 0 (0 to 0) | -2.14% (-2.51 to -1.76) |
| Djibouti | 0 (0 to 0) | 0.01 (0 to 0.02) | 0 (0 to 0) | 0.01 (0 to 0.02) | -0.16% (-0.57 to 0.25) |
| Dominica | 0 (0 to 0) | 0 (0 to 0) | 0 (0 to 0) | 0 (0 to 0) | 5.03% (4.68 to 5.38) |
| Dominican Republic | 0 (0 to 0) | 0 (0 to 0) | 0 (0 to 0) | 0 (0 to 0) | 0.35% (0.12 to 0.58) |
| Ecuador | 0 (0 to 0) | 0 (0 to 0) | 0 (0 to 0) | 0 (0 to 0) | 1.44% (0.52 to 2.36) |
| Egypt | 1 (0 to 1) | 0 (0 to 0.01) | 1 (0 to 2) | 0 (0 to 0) | 0.19% (-0.42 to 0.81) |
| El Salvador | 0 (0 to 0) | 0 (0 to 0) | 0 (0 to 0) | 0 (0 to 0) | -0.96% (-1.43 to -0.5) |
| Equatorial Guinea | 0 (0 to 0) | 0 (0 to 0.01) | 0 (0 to 0) | 0 (0 to 0) | -1.43% (-1.68 to -1.18) |
| Eritrea | 0 (0 to 0) | 0.01 (0 to 0.03) | 0 (0 to 0) | 0.01 (0 to 0.02) | -0.94% (-1.11 to -0.77) |
| Estonia | 0 (0 to 0) | 0 (0 to 0.01) | 0 (0 to 0) | 0 (0 to 0) | -2.49% (-3.23 to -1.73) |
| Eswatini | 0 (0 to 0) | 0 (0 to 0.01) | 0 (0 to 0) | 0.01 (0 to 0.01) | 1.37% (0.81 to 1.94) |
| Ethiopia | 3 (1 to 8) | 0.02 (0 to 0.04) | 3 (1 to 7) | 0.01 (0 to 0.02) | -3.06% (-3.25 to -2.87) |
| Fiji | 0 (0 to 0) | 0 (0 to 0) | 0 (0 to 0) | 0 (0 to 0) | 2.65% (2.3 to 3) |
| Finland | 0 (0 to 0) | 0 (0 to 0) | 0 (0 to 0) | 0 (0 to 0) | -0.56% (-0.93 to -0.19) |
| France | 0 (0 to 0) | 0 (0 to 0) | 0 (0 to 0) | 0 (0 to 0) | -2.48% (-2.71 to -2.25) |
| Gabon | 0 (0 to 0) | 0 (0 to 0) | 0 (0 to 0) | 0 (0 to 0) | -0.21% (-0.4 to -0.03) |
| Gambia | 0 (0 to 0) | 0 (0 to 0.01) | 0 (0 to 0) | 0 (0 to 0.01) | -0.81% (-1.12 to -0.51) |
| Georgia | 0 (0 to 0) | 0 (0 to 0) | 0 (0 to 0) | 0 (0 to 0) | 1.27% (0.4 to 2.15) |
| Germany | 0 (0 to 0) | 0 (0 to 0) | 0 (0 to 0) | 0 (0 to 0) | -1.14% (-1.5 to -0.77) |
| Ghana | 0 (0 to 1) | 0.01 (0 to 0.01) | 1 (0 to 1) | 0.01 (0 to 0.01) | -0.06% (-0.22 to 0.1) |
| Greece | 0 (0 to 0) | 0 (0 to 0) | 0 (0 to 0) | 0 (0 to 0) | -1.54% (-1.98 to -1.09) |
| Greenland | 0 (0 to 0) | 0 (0 to 0) | 0 (0 to 0) | 0 (0 to 0) | -3.27% (-3.8 to -2.73) |
| Grenada | 0 (0 to 0) | 0 (0 to 0) | 0 (0 to 0) | 0 (0 to 0) | 2.38% (1.8 to 2.96) |
| Guam | 0 (0 to 0) | 0 (0 to 0) | 0 (0 to 0) | 0 (0 to 0) | -5.54% (-6.56 to -4.51) |
| Guatemala | 0 (0 to 1) | 0.01 (0.01 to 0.01) | 0 (0 to 0) | 0 (0 to 0.01) | -1.99% (-2.51 to -1.48) |
| Guinea | 0 (0 to 0) | 0.01 (0 to 0.01) | 0 (0 to 0) | 0 (0 to 0.01) | -0.91% (-1.03 to -0.8) |
| Guinea-Bissau | 0 (0 to 0) | 0.01 (0 to 0.02) | 0 (0 to 0) | 0.01 (0 to 0.01) | -1.74% (-1.88 to -1.61) |
| Guyana | 0 (0 to 0) | 0 (0 to 0) | 0 (0 to 0) | 0 (0 to 0) | 4.7% (3.51 to 5.9) |
| Haiti | 0 (0 to 0) | 0 (0 to 0) | 0 (0 to 0) | 0 (0 to 0) | 0.4% (0.19 to 0.6) |
| Honduras | 0 (0 to 0) | 0.01 (0.01 to 0.03) | 0 (0 to 0) | 0 (0 to 0.01) | -4.08% (-4.3 to -3.85) |
| Hungary | 0 (0 to 1) | 0.02 (0.01 to 0.02) | 0 (0 to 0) | 0 (0 to 0) | -6.72% (-7.65 to -5.78) |
| Iceland | 0 (0 to 0) | 0 (0 to 0) | 0 (0 to 0) | 0 (0 to 0) | -0.18% (-0.64 to 0.28) |
| India | 20 (5 to 36) | 0.01 (0 to 0.01) | 9 (4 to 15) | 0 (0 to 0) | -3.65% (-3.9 to -3.4) |
| Indonesia | 3 (0 to 7) | 0.01 (0 to 0.01) | 3 (0 to 5) | 0 (0 to 0.01) | -1.17% (-1.34 to -1) |
| Iran (Islamic Republic of) | 8 (2 to 13) | 0.03 (0.01 to 0.06) | 2 (1 to 4) | 0.01 (0 to 0.02) | -2.99% (-3.74 to -2.23) |
| Iraq | 0 (0 to 1) | 0.01 (0 to 0.01) | 0 (0 to 1) | 0 (0 to 0) | -3.11% (-3.59 to -2.63) |
| Ireland | 0 (0 to 0) | 0 (0 to 0) | 0 (0 to 0) | 0 (0 to 0) | -2.69% (-3.19 to -2.19) |
| Israel | 0 (0 to 0) | 0 (0 to 0) | 0 (0 to 0) | 0 (0 to 0) | -1.58% (-1.92 to -1.23) |
| Italy | 0 (0 to 0) | 0 (0 to 0) | 0 (0 to 0) | 0 (0 to 0) | -4.74% (-5.35 to -4.13) |
| Jamaica | 0 (0 to 0) | 0 (0 to 0) | 0 (0 to 0) | 0 (0 to 0) | 1.66% (0.86 to 2.47) |
| Japan | 0 (0 to 0) | 0 (0 to 0) | 0 (0 to 0) | 0 (0 to 0) | 1.96% (1.8 to 2.12) |
| Jordan | 0 (0 to 0) | 0 (0 to 0) | 0 (0 to 0) | 0 (0 to 0) | 0.85% (0.63 to 1.07) |
| Kazakhstan | 1 (0 to 1) | 0.02 (0.01 to 0.02) | 0 (0 to 1) | 0.01 (0.01 to 0.01) | -1.44% (-1.91 to -0.96) |
| Kenya | 0 (0 to 1) | 0 (0 to 0.01) | 1 (0 to 2) | 0.01 (0 to 0.01) | 1.19% (0.9 to 1.48) |
| Kiribati | 0 (0 to 0) | 0 (0 to 0) | 0 (0 to 0) | 0 (0 to 0) | -1.92% (-2.08 to -1.76) |
| Kuwait | 0 (0 to 0) | 0 (0 to 0) | 0 (0 to 0) | 0 (0 to 0) | 20.66% (17.36 to 24.05) |
| Kyrgyzstan | 0 (0 to 0) | 0.01 (0 to 0.01) | 0 (0 to 0) | 0 (0 to 0) | -2.4% (-2.83 to -1.96) |
| Lao People's Democratic Republic | 0 (0 to 0) | 0.01 (0 to 0.02) | 0 (0 to 0) | 0 (0 to 0.01) | -2.8% (-2.97 to -2.64) |
| Latvia | 0 (0 to 0) | 0.01 (0 to 0.01) | 0 (0 to 0) | 0 (0 to 0) | -2.55% (-3.23 to -1.86) |
| Lebanon | 0 (0 to 0) | 0 (0 to 0.01) | 0 (0 to 0) | 0 (0 to 0) | -2.51% (-2.63 to -2.39) |
| Lesotho | 0 (0 to 0) | 0 (0 to 0.01) | 0 (0 to 0) | 0.01 (0 to 0.01) | 3.6% (3.17 to 4.04) |
| Liberia | 0 (0 to 0) | 0.01 (0 to 0.01) | 0 (0 to 0) | 0 (0 to 0.01) | -1.55% (-2.07 to -1.02) |
| Libya | 0 (0 to 0) | 0 (0 to 0) | 0 (0 to 0) | 0.01 (0 to 0.01) | 6.82% (6.09 to 7.56) |
| Lithuania | 0 (0 to 0) | 0 (0 to 0.01) | 0 (0 to 0) | 0 (0 to 0) | -1.52% (-2.03 to -1.01) |
| Luxembourg | 0 (0 to 0) | 0 (0 to 0) | 0 (0 to 0) | 0 (0 to 0) | -3.42% (-3.95 to -2.88) |
| Madagascar | 0 (0 to 1) | 0.01 (0 to 0.02) | 1 (0 to 1) | 0 (0 to 0.01) | -0.83% (-0.97 to -0.7) |
| Malawi | 0 (0 to 1) | 0.01 (0 to 0.02) | 0 (0 to 1) | 0.01 (0 to 0.01) | -0.82% (-1.01 to -0.62) |
| Malaysia | 0 (0 to 0) | 0 (0 to 0) | 0 (0 to 0) | 0 (0 to 0) | -1.01% (-1.31 to -0.7) |
| Maldives | 0 (0 to 0) | 0 (0 to 0) | 0 (0 to 0) | 0 (0 to 0) | -2.51% (-2.81 to -2.21) |
| Mali | 0 (0 to 0) | 0.01 (0 to 0.01) | 0 (0 to 1) | 0 (0 to 0.01) | -1.25% (-1.35 to -1.14) |
| Malta | 0 (0 to 0) | 0 (0 to 0) | 0 (0 to 0) | 0 (0 to 0) | -0.98% (-1.81 to -0.13) |
| Marshall Islands | 0 (0 to 0) | 0 (0 to 0) | 0 (0 to 0) | 0 (0 to 0) | -0.43% (-0.85 to -0.01) |
| Mauritania | 0 (0 to 0) | 0.01 (0 to 0.01) | 0 (0 to 0) | 0 (0 to 0.01) | -2.35% (-2.43 to -2.27) |
| Mauritius | 0 (0 to 0) | 0 (0 to 0) | 0 (0 to 0) | 0 (0 to 0) | 2.97% (0.96 to 5.03) |
| Mexico | 3 (3 to 3) | 0.01 (0.01 to 0.01) | 2 (1 to 2) | 0 (0 to 0.01) | -1.41% (-1.66 to -1.16) |
| Micronesia (Federated States of) | 0 (0 to 0) | 0 (0 to 0) | 0 (0 to 0) | 0 (0 to 0) | -2.4% (-2.53 to -2.28) |
| Monaco | 0 (0 to 0) | 0 (0 to 0) | 0 (0 to 0) | 0 (0 to 0) | -0.59% (-0.98 to -0.2) |
| Mongolia | 0 (0 to 0) | 0 (0 to 0) | 0 (0 to 0) | 0 (0 to 0) | -0.86% (-1.73 to 0.02) |
| Montenegro | 0 (0 to 0) | 0 (0 to 0) | 0 (0 to 0) | 0 (0 to 0) | -3.27% (-4.32 to -2.2) |
| Morocco | 0 (0 to 0) | 0 (0 to 0) | 0 (0 to 1) | 0 (0 to 0.01) | 3.02% (2.01 to 4.04) |
| Mozambique | 0 (0 to 1) | 0.01 (0 to 0.02) | 1 (0 to 2) | 0.01 (0 to 0.02) | 0.88% (0.62 to 1.14) |
| Myanmar | 1 (0 to 4) | 0.01 (0 to 0.03) | 1 (0 to 2) | 0 (0 to 0.01) | -3.04% (-3.4 to -2.69) |
| Namibia | 0 (0 to 0) | 0 (0 to 0.01) | 0 (0 to 0) | 0 (0 to 0.01) | -0.01% (-0.33 to 0.3) |
| Nauru | 0 (0 to 0) | 0 (0 to 0) | 0 (0 to 0) | 0 (0 to 0) | -1.32% (-1.66 to -0.99) |
| Nepal | 0 (0 to 1) | 0 (0 to 0.01) | 0 (0 to 0) | 0 (0 to 0.01) | -1.93% (-2.21 to -1.65) |
| Netherlands | 0 (0 to 0) | 0 (0 to 0) | 0 (0 to 0) | 0 (0 to 0) | -1.85% (-2.23 to -1.47) |
| New Zealand | 0 (0 to 0) | 0 (0 to 0) | 0 (0 to 0) | 0 (0 to 0) | -1.9% (-2.68 to -1.11) |
| Nicaragua | 0 (0 to 0) | 0 (0 to 0) | 0 (0 to 0) | 0 (0 to 0) | -2.04% (-2.29 to -1.79) |
| Niger | 0 (0 to 0) | 0.01 (0 to 0.01) | 0 (0 to 1) | 0 (0 to 0.01) | -2.8% (-3.03 to -2.58) |
| Nigeria | 2 (1 to 3) | 0 (0 to 0.01) | 3 (2 to 6) | 0 (0 to 0.01) | -0.84% (-0.95 to -0.73) |
| Niue | 0 (0 to 0) | 0 (0 to 0) | 0 (0 to 0) | 0 (0 to 0) | 0.33% (-0.59 to 1.25) |
| North Macedonia | 0 (0 to 0) | 0 (0 to 0) | 0 (0 to 0) | 0 (0 to 0) | -4.62% (-5.46 to -3.78) |
| Northern Mariana Islands | 0 (0 to 0) | 0 (0 to 0) | 0 (0 to 0) | 0 (0 to 0) | -3.11% (-4.72 to -1.47) |
| Norway | 0 (0 to 0) | 0 (0 to 0) | 0 (0 to 0) | 0 (0 to 0) | -2.69% (-3.25 to -2.13) |
| Oman | 0 (0 to 0) | 0 (0 to 0) | 0 (0 to 0) | 0 (0 to 0) | 2.93% (2.03 to 3.84) |
| Pakistan | 2 (1 to 5) | 0.01 (0 to 0.01) | 5 (1 to 9) | 0.01 (0 to 0.01) | 0.53% (0.27 to 0.79) |
| Palau | 0 (0 to 0) | 0 (0 to 0) | 0 (0 to 0) | 0 (0 to 0) | -2.33% (-2.4 to -2.27) |
| Palestine | 0 (0 to 0) | 0 (0 to 0) | 0 (0 to 0) | 0 (0 to 0) | -1.51% (-2.21 to -0.81) |
| Panama | 0 (0 to 0) | 0 (0 to 0) | 0 (0 to 0) | 0 (0 to 0) | -0.65% (-0.92 to -0.37) |
| Papua New Guinea | 0 (0 to 0) | 0 (0 to 0) | 0 (0 to 0) | 0 (0 to 0) | -1.88% (-2.02 to -1.74) |
| Paraguay | 0 (0 to 0) | 0 (0 to 0) | 0 (0 to 0) | 0 (0 to 0.01) | 4.55% (4.2 to 4.91) |
| Peru | 0 (0 to 0) | 0 (0 to 0) | 0 (0 to 0) | 0 (0 to 0) | -0.48% (-0.72 to -0.23) |
| Philippines | 4 (2 to 7) | 0.02 (0.01 to 0.03) | 4 (2 to 6) | 0.01 (0.01 to 0.02) | -0.89% (-1.15 to -0.64) |
| Poland | 0 (0 to 0) | 0 (0 to 0) | 0 (0 to 0) | 0 (0 to 0) | -7.81% (-9.33 to -6.26) |
| Portugal | 0 (0 to 0) | 0 (0 to 0) | 0 (0 to 0) | 0 (0 to 0) | -3.9% (-4.27 to -3.53) |
| Puerto Rico | 0 (0 to 0) | 0 (0 to 0) | 0 (0 to 0) | 0 (0 to 0) | 2.52% (1.41 to 3.65) |
| Qatar | 0 (0 to 0) | 0 (0 to 0) | 0 (0 to 0) | 0 (0 to 0) | -4.35% (-5.09 to -3.6) |
| Republic of Korea | 0 (0 to 0) | 0 (0 to 0) | 0 (0 to 0) | 0 (0 to 0) | -3.85% (-4.49 to -3.2) |
| Republic of Moldova | 0 (0 to 0) | 0 (0 to 0) | 0 (0 to 0) | 0 (0 to 0) | -1.1% (-1.62 to -0.58) |
| Romania | 0 (0 to 0) | 0 (0 to 0) | 0 (0 to 0) | 0 (0 to 0) | -5.98% (-6.38 to -5.58) |
| Russian Federation | 3 (2 to 3) | 0.01 (0.01 to 0.01) | 1 (1 to 1) | 0 (0 to 0) | -2.13% (-2.62 to -1.64) |
| Rwanda | 0 (0 to 1) | 0.01 (0 to 0.03) | 0 (0 to 1) | 0 (0 to 0.01) | -3.53% (-3.86 to -3.21) |
| Saint Kitts and Nevis | 0 (0 to 0) | 0 (0 to 0) | 0 (0 to 0) | 0 (0 to 0) | 3.57% (2.74 to 4.4) |
| Saint Lucia | 0 (0 to 0) | 0 (0 to 0) | 0 (0 to 0) | 0 (0 to 0) | 3.6% (2.63 to 4.58) |
| Saint Vincent and the Grenadines | 0 (0 to 0) | 0 (0 to 0) | 0 (0 to 0) | 0 (0 to 0) | -0.19% (-0.75 to 0.37) |
| Samoa | 0 (0 to 0) | 0 (0 to 0) | 0 (0 to 0) | 0 (0 to 0) | -2.55% (-2.69 to -2.41) |
| San Marino | 0 (0 to 0) | 0 (0 to 0) | 0 (0 to 0) | 0 (0 to 0) | -1% (-1.28 to -0.71) |
| Sao Tome and Principe | 0 (0 to 0) | 0.01 (0 to 0.01) | 0 (0 to 0) | 0 (0 to 0.01) | -2.2% (-2.45 to -1.94) |
| Saudi Arabia | 0 (0 to 0) | 0 (0 to 0) | 0 (0 to 0) | 0 (0 to 0) | 2.02% (0.99 to 3.07) |
| Senegal | 0 (0 to 0) | 0.01 (0 to 0.01) | 0 (0 to 0) | 0 (0 to 0.01) | -1.28% (-1.46 to -1.11) |
| Serbia | 0 (0 to 0) | 0 (0 to 0) | 0 (0 to 0) | 0 (0 to 0) | -5.67% (-6.06 to -5.28) |
| Seychelles | 0 (0 to 0) | 0 (0 to 0) | 0 (0 to 0) | 0 (0 to 0) | 1.3% (-0.62 to 3.27) |
| Sierra Leone | 0 (0 to 0) | 0.01 (0 to 0.01) | 0 (0 to 0) | 0 (0 to 0.01) | -1.05% (-1.21 to -0.9) |
| Singapore | 0 (0 to 0) | 0 (0 to 0) | 0 (0 to 0) | 0 (0 to 0) | 0.52% (0.09 to 0.95) |
| Slovakia | 0 (0 to 0) | 0 (0 to 0) | 0 (0 to 0) | 0 (0 to 0) | -4.32% (-4.78 to -3.87) |
| Slovenia | 0 (0 to 0) | 0 (0 to 0) | 0 (0 to 0) | 0 (0 to 0) | -6.96% (-8.09 to -5.82) |
| Solomon Islands | 0 (0 to 0) | 0 (0 to 0) | 0 (0 to 0) | 0 (0 to 0) | -1.4% (-1.55 to -1.25) |
| Somalia | 0 (0 to 1) | 0.01 (0 to 0.03) | 1 (0 to 2) | 0.01 (0 to 0.03) | -0.13% (-0.41 to 0.16) |
| South Africa | 0 (0 to 0) | 0 (0 to 0) | 0 (0 to 1) | 0 (0 to 0) | -0.65% (-1.28 to -0.02) |
| South Sudan | 0 (0 to 1) | 0.01 (0 to 0.02) | 0 (0 to 1) | 0.01 (0 to 0.03) | 0.56% (0.18 to 0.95) |
| Spain | 0 (0 to 0) | 0 (0 to 0) | 0 (0 to 0) | 0 (0 to 0) | -3.02% (-3.33 to -2.72) |
| Sri Lanka | 0 (0 to 0) | 0 (0 to 0) | 0 (0 to 0) | 0 (0 to 0) | -2.33% (-2.8 to -1.84) |
| Sudan | 0 (0 to 0) | 0 (0 to 0.01) | 1 (0 to 2) | 0.01 (0 to 0.01) | 4.15% (3.42 to 4.89) |
| Suriname | 0 (0 to 0) | 0 (0 to 0) | 0 (0 to 0) | 0 (0 to 0) | 1.61% (1.16 to 2.06) |
| Sweden | 0 (0 to 0) | 0 (0 to 0) | 0 (0 to 0) | 0 (0 to 0) | -1.42% (-2.18 to -0.65) |
| Switzerland | 0 (0 to 0) | 0 (0 to 0) | 0 (0 to 0) | 0 (0 to 0) | -2.59% (-2.86 to -2.32) |
| Syrian Arab Republic | 0 (0 to 1) | 0.01 (0 to 0.02) | 0 (0 to 0) | 0 (0 to 0.01) | -3.19% (-3.71 to -2.66) |
| Taiwan (Province of China) | 0 (0 to 0) | 0 (0 to 0.01) | 0 (0 to 0) | 0 (0 to 0) | -2.64% (-3.55 to -1.73) |
| Tajikistan | 0 (0 to 0) | 0.01 (0 to 0.02) | 0 (0 to 1) | 0.01 (0 to 0.02) | -1.73% (-2.17 to -1.29) |
| Thailand | 3 (1 to 6) | 0.02 (0.01 to 0.03) | 1 (0 to 2) | 0.01 (0 to 0.02) | -2.91% (-3.24 to -2.58) |
| Timor-Leste | 0 (0 to 0) | 0.01 (0 to 0.01) | 0 (0 to 0) | 0 (0 to 0.01) | -2.07% (-2.73 to -1.4) |
| Togo | 0 (0 to 0) | 0 (0 to 0.01) | 0 (0 to 0) | 0 (0 to 0.01) | -0.93% (-1.07 to -0.79) |
| Tokelau | 0 (0 to 0) | 0 (0 to 0) | 0 (0 to 0) | 0 (0 to 0) | -0.96% (-2.15 to 0.25) |
| Tonga | 0 (0 to 0) | 0 (0 to 0) | 0 (0 to 0) | 0 (0 to 0) | -0.17% (-0.56 to 0.22) |
| Trinidad and Tobago | 0 (0 to 0) | 0 (0 to 0) | 0 (0 to 0) | 0 (0 to 0) | 1.91% (1.36 to 2.47) |
| Tunisia | 0 (0 to 0) | 0 (0 to 0) | 0 (0 to 0) | 0 (0 to 0) | 2.18% (1.43 to 2.93) |
| Turkey | 1 (0 to 2) | 0 (0 to 0.01) | 0 (0 to 1) | 0 (0 to 0) | -3.34% (-3.62 to -3.06) |
| Turkmenistan | 0 (0 to 0) | 0 (0 to 0) | 0 (0 to 0) | 0 (0 to 0.01) | -0.3% (-0.57 to -0.03) |
| Tuvalu | 0 (0 to 0) | 0 (0 to 0) | 0 (0 to 0) | 0 (0 to 0) | -2.8% (-2.87 to -2.72) |
| Uganda | 0 (0 to 1) | 0 (0 to 0.01) | 1 (0 to 2) | 0.01 (0 to 0.01) | -0.26% (-0.59 to 0.07) |
| Ukraine | 1 (0 to 1) | 0 (0 to 0.01) | 0 (0 to 0) | 0 (0 to 0) | -4.21% (-4.95 to -3.45) |
| United Arab Emirates | 0 (0 to 0) | 0 (0 to 0) | 0 (0 to 0) | 0 (0 to 0) | -1.25% (-1.77 to -0.72) |
| United Kingdom | 0 (0 to 0) | 0 (0 to 0) | 0 (0 to 0) | 0 (0 to 0) | -0.35% (-0.73 to 0.04) |
| United Republic of Tanzania | 1 (0 to 2) | 0.01 (0 to 0.02) | 1 (0 to 3) | 0.01 (0 to 0.02) | -0.92% (-1 to -0.84) |
| United States of America | 1 (1 to 1) | 0 (0 to 0) | 1 (1 to 1) | 0 (0 to 0) | 0.95% (0.51 to 1.38) |
| United States Virgin Islands | 0 (0 to 0) | 0 (0 to 0) | 0 (0 to 0) | 0 (0 to 0) | -2.28% (-3.6 to -0.95) |
| Uruguay | 0 (0 to 0) | 0 (0 to 0) | 0 (0 to 0) | 0 (0 to 0) | 1.29% (0.53 to 2.05) |
| Uzbekistan | 0 (0 to 0) | 0 (0 to 0) | 0 (0 to 0) | 0 (0 to 0) | 0.23% (-0.38 to 0.84) |
| Vanuatu | 0 (0 to 0) | 0 (0 to 0) | 0 (0 to 0) | 0 (0 to 0) | -1.08% (-1.29 to -0.86) |
| Venezuela (Bolivarian Republic of) | 0 (0 to 0) | 0.01 (0 to 0.01) | 1 (0 to 1) | 0.01 (0.01 to 0.01) | 1.86% (1.56 to 2.17) |
| Viet Nam | 0 (0 to 0) | 0 (0 to 0) | 0 (0 to 0) | 0 (0 to 0) | -0.45% (-0.71 to -0.19) |
| Yemen | 0 (0 to 0) | 0 (0 to 0) | 0 (0 to 1) | 0 (0 to 0.01) | 3.59% (2.8 to 4.39) |
| Zambia | 0 (0 to 1) | 0.01 (0 to 0.02) | 0 (0 to 1) | 0.01 (0 to 0.02) | -0.82% (-1.13 to -0.51) |
| Zimbabwe | 0 (0 to 0) | 0 (0 to 0) | 0 (0 to 0) | 0 (0 to 0.01) | 3.36% (2.84 to 3.88) |

***Table S3. pediatric stone disease DALYs for both sexes and for 204 countries in 1990 and 2021, and estimated annual percentage changes from 1990 to 2021.***

| location | Num_1990 | ASR_1990 | Num_2021 | ASR_2021 | EAPC_CI |
| --- | --- | --- | --- | --- | --- |
| Afghanistan | 24 (12 to 41) | 0.62 (0.32 to 1.06) | 125 (47 to 244) | 1.02 (0.39 to 1.99) | 2.23% (1.79 to 2.68) |
| Albania | 14 (8 to 23) | 1.35 (0.77 to 2.22) | 5 (3 to 9) | 1.05 (0.53 to 1.87) | -0.77% (-0.82 to -0.72) |
| Algeria | 57 (30 to 98) | 0.59 (0.31 to 1.02) | 79 (36 to 144) | 0.68 (0.31 to 1.24) | 0.96% (0.69 to 1.23) |
| American Samoa | 0 (0 to 0) | 0.26 (0.12 to 0.49) | 0 (0 to 0) | 0.27 (0.12 to 0.49) | 0.08% (0.05 to 0.11) |
| Andorra | 0 (0 to 0) | 0.34 (0.18 to 0.57) | 0 (0 to 0) | 0.26 (0.13 to 0.46) | -0.73% (-0.85 to -0.61) |
| Angola | 20 (10 to 37) | 0.54 (0.26 to 0.99) | 58 (31 to 100) | 0.47 (0.25 to 0.8) | -0.45% (-0.48 to -0.42) |
| Antigua and Barbuda | 0 (0 to 0) | 0.37 (0.17 to 0.68) | 0 (0 to 0) | 0.39 (0.18 to 0.7) | 0.18% (0.14 to 0.22) |
| Argentina | 44 (22 to 78) | 0.46 (0.23 to 0.82) | 54 (27 to 94) | 0.48 (0.25 to 0.85) | 0.13% (0.07 to 0.2) |
| Armenia | 19 (11 to 29) | 2.05 (1.2 to 3.06) | 10 (6 to 16) | 1.75 (1.07 to 2.77) | 0% (-0.4 to 0.4) |
| Australia | 9 (4 to 16) | 0.21 (0.09 to 0.38) | 10 (4 to 18) | 0.2 (0.09 to 0.38) | -0.06% (-0.08 to -0.04) |
| Austria | 8 (6 to 12) | 0.53 (0.37 to 0.75) | 5 (3 to 8) | 0.37 (0.23 to 0.59) | -1% (-1.18 to -0.81) |
| Azerbaijan | 26 (14 to 44) | 1.17 (0.62 to 1.95) | 26 (14 to 45) | 1.14 (0.6 to 1.95) | -0.17% (-0.24 to -0.1) |
| Bahamas | 0 (0 to 1) | 0.37 (0.18 to 0.67) | 0 (0 to 1) | 0.39 (0.2 to 0.69) | 0.16% (0.11 to 0.22) |
| Bahrain | 1 (0 to 1) | 0.44 (0.22 to 0.77) | 2 (1 to 3) | 0.52 (0.27 to 0.92) | 0.74% (0.57 to 0.91) |
| Bangladesh | 463 (197 to 855) | 1.12 (0.49 to 2.05) | 382 (205 to 638) | 0.81 (0.43 to 1.35) | -0.9% (-1.01 to -0.79) |
| Barbados | 0 (0 to 0) | 0.39 (0.19 to 0.7) | 0 (0 to 0) | 0.41 (0.21 to 0.72) | 0.22% (0.15 to 0.29) |
| Belarus | 30 (17 to 48) | 1.28 (0.73 to 2.05) | 16 (9 to 28) | 1.09 (0.6 to 1.83) | -0.54% (-0.59 to -0.49) |
| Belgium | 6 (4 to 10) | 0.3 (0.18 to 0.5) | 5 (3 to 9) | 0.26 (0.14 to 0.46) | 0.06% (-0.5 to 0.61) |
| Belize | 0 (0 to 0) | 0.38 (0.18 to 0.69) | 1 (0 to 1) | 0.42 (0.22 to 0.72) | 0.38% (0.32 to 0.44) |
| Benin | 14 (8 to 24) | 0.79 (0.44 to 1.32) | 33 (18 to 53) | 0.64 (0.36 to 1.05) | -0.62% (-0.67 to -0.57) |
| Bermuda | 0 (0 to 0) | 0.37 (0.17 to 0.68) | 0 (0 to 0) | 0.38 (0.18 to 0.68) | 0.15% (0.11 to 0.19) |
| Bhutan | 2 (1 to 4) | 0.91 (0.44 to 1.72) | 2 (1 to 3) | 0.75 (0.39 to 1.34) | -0.68% (-0.77 to -0.6) |
| Bolivia (Plurinational State of) | 12 (7 to 20) | 0.54 (0.3 to 0.9) | 16 (9 to 28) | 0.48 (0.26 to 0.82) | -0.42% (-0.44 to -0.41) |
| Bosnia and Herzegovina | 14 (8 to 24) | 1.22 (0.68 to 2.03) | 6 (3 to 10) | 1.06 (0.56 to 1.84) | -0.51% (-0.58 to -0.43) |
| Botswana | 4 (2 to 6) | 0.7 (0.37 to 1.2) | 5 (3 to 8) | 0.7 (0.36 to 1.23) | 0.22% (0.02 to 0.42) |
| Brazil | 231 (169 to 325) | 0.46 (0.34 to 0.64) | 485 (390 to 620) | 0.98 (0.78 to 1.25) | 2.83% (2.5 to 3.15) |
| Brunei Darussalam | 0 (0 to 1) | 0.54 (0.3 to 0.88) | 0 (0 to 1) | 0.41 (0.22 to 0.71) | -0.71% (-0.81 to -0.6) |
| Bulgaria | 42 (31 to 60) | 2.23 (1.63 to 3.16) | 12 (6 to 20) | 1.15 (0.64 to 1.96) | -1.9% (-2.25 to -1.55) |
| Burkina Faso | 26 (15 to 44) | 0.73 (0.4 to 1.21) | 54 (29 to 89) | 0.64 (0.35 to 1.05) | -0.28% (-0.35 to -0.2) |
| Burundi | 23 (9 to 47) | 1.17 (0.48 to 2.46) | 40 (21 to 74) | 0.83 (0.43 to 1.52) | -1.25% (-1.34 to -1.16) |
| Cabo Verde | 1 (0 to 1) | 0.46 (0.25 to 0.79) | 1 (0 to 1) | 0.49 (0.27 to 0.85) | 0% (-0.11 to 0.11) |
| Cambodia | 33 (10 to 69) | 0.9 (0.28 to 1.85) | 29 (11 to 54) | 0.59 (0.22 to 1.11) | -1.7% (-1.84 to -1.55) |
| Cameroon | 32 (18 to 53) | 0.84 (0.48 to 1.38) | 82 (47 to 139) | 0.7 (0.39 to 1.18) | -0.6% (-0.71 to -0.5) |
| Canada | 28 (15 to 46) | 0.46 (0.26 to 0.78) | 30 (17 to 52) | 0.46 (0.26 to 0.79) | 0.16% (0.09 to 0.23) |
| Central African Republic | 5 (3 to 10) | 0.55 (0.27 to 0.98) | 11 (6 to 18) | 0.53 (0.28 to 0.89) | -0.11% (-0.14 to -0.08) |
| Chad | 15 (8 to 25) | 0.68 (0.37 to 1.16) | 48 (27 to 81) | 0.68 (0.38 to 1.15) | 0.07% (-0.03 to 0.16) |
| Chile | 19 (10 to 34) | 0.48 (0.25 to 0.84) | 25 (16 to 40) | 0.65 (0.4 to 1.02) | 0.64% (0.27 to 1) |
| China | 4296 (1863 to 6220) | 1.26 (0.54 to 1.83) | 717 (441 to 1107) | 0.28 (0.17 to 0.44) | -5.44% (-5.71 to -5.17) |
| Colombia | 61 (40 to 94) | 0.56 (0.36 to 0.86) | 53 (32 to 86) | 0.46 (0.27 to 0.75) | -0.68% (-0.77 to -0.59) |
| Comoros | 2 (1 to 3) | 0.92 (0.45 to 1.66) | 2 (1 to 4) | 0.84 (0.42 to 1.64) | -0.69% (-1.1 to -0.28) |
| Congo | 5 (2 to 8) | 0.51 (0.26 to 0.89) | 9 (5 to 15) | 0.47 (0.25 to 0.8) | -0.26% (-0.32 to -0.21) |
| Cook Islands | 0 (0 to 0) | 0.26 (0.12 to 0.49) | 0 (0 to 0) | 0.27 (0.12 to 0.49) | 0.06% (0.04 to 0.08) |
| Costa Rica | 4 (2 to 7) | 0.38 (0.2 to 0.66) | 4 (2 to 7) | 0.36 (0.18 to 0.64) | -0.09% (-0.12 to -0.06) |
| Coted'Ivoire | 33 (19 to 54) | 0.74 (0.43 to 1.21) | 66 (38 to 107) | 0.67 (0.39 to 1.09) | -0.29% (-0.39 to -0.19) |
| Croatia | 13 (7 to 21) | 1.22 (0.69 to 2.03) | 5 (3 to 9) | 0.77 (0.41 to 1.31) | -1.58% (-2.36 to -0.8) |
| Cuba | 12 (6 to 22) | 0.39 (0.19 to 0.7) | 8 (4 to 14) | 0.4 (0.2 to 0.72) | 0.14% (0.11 to 0.17) |
| Cyprus | 1 (0 to 1) | 0.28 (0.15 to 0.46) | 0 (0 to 1) | 0.2 (0.1 to 0.36) | -1.22% (-1.63 to -0.81) |
| Czechia | 45 (31 to 67) | 1.79 (1.23 to 2.65) | 18 (10 to 32) | 1.11 (0.57 to 1.9) | -1.65% (-2.07 to -1.22) |
| Democratic People's Republic of Korea | 43 (18 to 83) | 0.76 (0.32 to 1.48) | 23 (12 to 43) | 0.42 (0.21 to 0.79) | -1.93% (-2.06 to -1.8) |
| Democratic Republic of the Congo | 68 (35 to 119) | 0.49 (0.25 to 0.85) | 154 (83 to 261) | 0.46 (0.25 to 0.77) | -0.15% (-0.18 to -0.12) |
| Denmark | 3 (2 to 5) | 0.31 (0.19 to 0.47) | 3 (1 to 4) | 0.25 (0.14 to 0.41) | -0.87% (-1.59 to -0.14) |
| Djibouti | 2 (1 to 3) | 0.99 (0.45 to 1.98) | 4 (2 to 7) | 0.97 (0.48 to 1.97) | -0.06% (-0.3 to 0.18) |
| Dominica | 0 (0 to 0) | 0.37 (0.17 to 0.68) | 0 (0 to 0) | 0.38 (0.18 to 0.68) | 0.12% (0.1 to 0.13) |
| Dominican Republic | 10 (5 to 17) | 0.38 (0.18 to 0.68) | 11 (5 to 20) | 0.37 (0.18 to 0.68) | -0.01% (-0.02 to 0.01) |
| Ecuador | 14 (8 to 23) | 0.39 (0.22 to 0.64) | 24 (16 to 36) | 0.48 (0.32 to 0.71) | 1.31% (1.07 to 1.55) |
| Egypt | 133 (68 to 229) | 0.69 (0.35 to 1.18) | 199 (115 to 327) | 0.6 (0.34 to 0.99) | 0.08% (-0.15 to 0.3) |
| El Salvador | 9 (5 to 15) | 0.47 (0.28 to 0.77) | 7 (4 to 13) | 0.42 (0.23 to 0.72) | -0.25% (-0.37 to -0.14) |
| Equatorial Guinea | 1 (0 to 1) | 0.53 (0.27 to 0.95) | 3 (2 to 5) | 0.5 (0.26 to 0.88) | -0.22% (-0.28 to -0.15) |
| Eritrea | 14 (6 to 31) | 1.12 (0.45 to 2.4) | 22 (11 to 42) | 0.97 (0.47 to 1.84) | -0.55% (-0.65 to -0.45) |
| Estonia | 4 (3 to 7) | 1.22 (0.73 to 1.94) | 2 (1 to 4) | 1.03 (0.57 to 1.73) | -0.53% (-0.65 to -0.42) |
| Eswatini | 3 (1 to 4) | 0.79 (0.43 to 1.35) | 4 (2 to 6) | 0.91 (0.48 to 1.57) | 0.71% (0.46 to 0.95) |
| Ethiopia | 307 (122 to 652) | 1.66 (0.66 to 3.52) | 402 (209 to 757) | 0.97 (0.5 to 1.83) | -1.92% (-2.07 to -1.78) |
| Fiji | 1 (0 to 1) | 0.25 (0.11 to 0.47) | 1 (0 to 1) | 0.27 (0.12 to 0.49) | 0.17% (0.16 to 0.18) |
| Finland | 2 (1 to 4) | 0.25 (0.14 to 0.41) | 2 (1 to 4) | 0.22 (0.12 to 0.38) | 0.21% (-0.67 to 1.11) |
| France | 36 (20 to 59) | 0.27 (0.15 to 0.44) | 29 (15 to 52) | 0.23 (0.11 to 0.4) | -0.48% (-0.54 to -0.43) |
| Gabon | 2 (1 to 3) | 0.49 (0.26 to 0.85) | 3 (2 to 5) | 0.48 (0.26 to 0.81) | -0.03% (-0.07 to 0.02) |
| Gambia | 3 (2 to 4) | 0.72 (0.41 to 1.15) | 6 (3 to 10) | 0.67 (0.37 to 1.14) | -0.37% (-0.51 to -0.23) |
| Georgia | 14 (7 to 23) | 1.02 (0.54 to 1.73) | 3 (2 to 5) | 0.51 (0.34 to 0.77) | -2.43% (-2.85 to -2.02) |
| Germany | 32 (17 to 54) | 0.24 (0.13 to 0.4) | 27 (14 to 47) | 0.22 (0.11 to 0.38) | 0.24% (-0.03 to 0.51) |
| Ghana | 45 (25 to 75) | 0.82 (0.46 to 1.38) | 91 (51 to 159) | 0.79 (0.45 to 1.38) | 0.05% (-0.02 to 0.13) |
| Greece | 5 (2 to 10) | 0.22 (0.1 to 0.41) | 5 (3 to 9) | 0.32 (0.16 to 0.58) | 1.95% (1.67 to 2.23) |
| Greenland | 0 (0 to 0) | 0.39 (0.19 to 0.71) | 0 (0 to 0) | 0.37 (0.18 to 0.67) | -0.13% (-0.15 to -0.11) |
| Grenada | 0 (0 to 0) | 0.38 (0.19 to 0.69) | 0 (0 to 0) | 0.42 (0.21 to 0.73) | 0.27% (0.23 to 0.31) |
| Guam | 0 (0 to 0) | 0.26 (0.12 to 0.49) | 0 (0 to 0) | 0.26 (0.11 to 0.48) | 0.03% (0 to 0.05) |
| Guatemala | 38 (25 to 54) | 1.12 (0.74 to 1.6) | 32 (20 to 47) | 0.61 (0.39 to 0.91) | -1.2% (-1.53 to -0.88) |
| Guinea | 16 (9 to 27) | 0.78 (0.42 to 1.31) | 33 (18 to 55) | 0.65 (0.36 to 1.08) | -0.45% (-0.5 to -0.4) |
| Guinea-Bissau | 4 (2 to 7) | 1.06 (0.54 to 1.89) | 6 (3 to 11) | 0.76 (0.4 to 1.42) | -1.05% (-1.13 to -0.98) |
| Guyana | 1 (1 to 2) | 0.39 (0.19 to 0.7) | 1 (1 to 2) | 0.44 (0.24 to 0.76) | 0.6% (0.48 to 0.72) |
| Haiti | 10 (5 to 18) | 0.45 (0.23 to 0.8) | 18 (9 to 32) | 0.45 (0.23 to 0.78) | 0.11% (0.07 to 0.15) |
| Honduras | 27 (13 to 45) | 1.42 (0.71 to 2.37) | 22 (12 to 36) | 0.65 (0.36 to 1.09) | -2.7% (-2.83 to -2.57) |
| Hungary | 56 (41 to 78) | 2.34 (1.69 to 3.22) | 17 (9 to 29) | 1.13 (0.61 to 1.93) | -1.81% (-2.2 to -1.43) |
| Iceland | 0 (0 to 0) | 0.24 (0.13 to 0.4) | 0 (0 to 0) | 0.21 (0.1 to 0.37) | 0.15% (-0.06 to 0.37) |
| India | 3494 (1723 to 5649) | 1.2 (0.6 to 1.94) | 3638 (2069 to 5951) | 0.89 (0.51 to 1.45) | -1.01% (-1.06 to -0.95) |
| Indonesia | 482 (180 to 782) | 0.74 (0.28 to 1.21) | 337 (114 to 538) | 0.47 (0.16 to 0.75) | -1.81% (-2.06 to -1.56) |
| Iran (Islamic Republic of) | 772 (251 to 1194) | 3.26 (1.07 to 5.05) | 289 (134 to 452) | 1.46 (0.68 to 2.29) | -2.35% (-2.94 to -1.76) |
| Iraq | 66 (34 to 116) | 0.91 (0.47 to 1.61) | 74 (39 to 128) | 0.55 (0.29 to 0.95) | -1.41% (-1.56 to -1.25) |
| Ireland | 3 (2 to 5) | 0.28 (0.16 to 0.46) | 2 (1 to 4) | 0.23 (0.11 to 0.4) | -0.57% (-0.68 to -0.47) |
| Israel | 5 (3 to 8) | 0.33 (0.21 to 0.51) | 6 (4 to 11) | 0.27 (0.15 to 0.44) | -0.58% (-0.7 to -0.46) |
| Italy | 53 (31 to 88) | 0.44 (0.26 to 0.72) | 28 (17 to 46) | 0.32 (0.19 to 0.52) | -1.43% (-1.61 to -1.25) |
| Jamaica | 3 (2 to 6) | 0.38 (0.19 to 0.68) | 3 (1 to 5) | 0.41 (0.21 to 0.72) | 0.18% (0.13 to 0.24) |
| Japan | 140 (70 to 247) | 0.48 (0.24 to 0.85) | 69 (39 to 113) | 0.4 (0.22 to 0.65) | -1.08% (-1.37 to -0.79) |
| Jordan | 7 (3 to 12) | 0.44 (0.22 to 0.8) | 21 (10 to 37) | 0.52 (0.26 to 0.93) | 0.78% (0.64 to 0.92) |
| Kazakhstan | 110 (69 to 161) | 2.32 (1.44 to 3.38) | 83 (52 to 125) | 1.75 (1.11 to 2.66) | -0.76% (-1.02 to -0.5) |
| Kenya | 72 (42 to 122) | 0.79 (0.46 to 1.34) | 162 (97 to 289) | 0.85 (0.51 to 1.52) | 0.55% (0.43 to 0.67) |
| Kiribati | 0 (0 to 0) | 0.27 (0.13 to 0.5) | 0 (0 to 0) | 0.28 (0.13 to 0.51) | 0.01% (0 to 0.02) |
| Kuwait | 2 (1 to 3) | 0.4 (0.19 to 0.73) | 4 (2 to 7) | 0.49 (0.27 to 0.84) | 0.85% (0.68 to 1.02) |
| Kyrgyzstan | 26 (15 to 40) | 1.77 (1.05 to 2.73) | 27 (16 to 46) | 1.39 (0.79 to 2.32) | -0.53% (-0.71 to -0.35) |
| Lao People's Democratic Republic | 15 (5 to 33) | 0.99 (0.3 to 2.18) | 13 (5 to 25) | 0.59 (0.23 to 1.12) | -1.87% (-1.97 to -1.78) |
| Latvia | 8 (5 to 12) | 1.42 (0.89 to 2.2) | 3 (2 to 5) | 1.03 (0.59 to 1.73) | -1.15% (-1.57 to -0.73) |
| Lebanon | 7 (4 to 12) | 0.72 (0.37 to 1.25) | 7 (4 to 12) | 0.55 (0.3 to 0.93) | -0.82% (-0.84 to -0.79) |
| Lesotho | 4 (2 to 6) | 0.66 (0.36 to 1.12) | 6 (3 to 10) | 0.92 (0.49 to 1.62) | 1.47% (1.33 to 1.61) |
| Liberia | 8 (4 to 13) | 0.87 (0.49 to 1.46) | 13 (7 to 21) | 0.64 (0.34 to 1.05) | -0.77% (-1.04 to -0.51) |
| Libya | 8 (4 to 14) | 0.5 (0.27 to 0.86) | 14 (6 to 26) | 0.86 (0.35 to 1.55) | 2.57% (2.28 to 2.87) |
| Lithuania | 12 (7 to 19) | 1.35 (0.82 to 2.18) | 5 (3 to 8) | 1.1 (0.63 to 1.85) | -0.7% (-1.05 to -0.36) |
| Luxembourg | 0 (0 to 0) | 0.27 (0.14 to 0.47) | 0 (0 to 0) | 0.24 (0.11 to 0.44) | -0.73% (-0.99 to -0.47) |
| Madagascar | 41 (19 to 82) | 0.94 (0.42 to 1.86) | 84 (42 to 158) | 0.79 (0.39 to 1.48) | -0.44% (-0.53 to -0.34) |
| Malawi | 33 (16 to 65) | 0.94 (0.46 to 1.84) | 65 (34 to 121) | 0.82 (0.43 to 1.52) | -0.44% (-0.53 to -0.34) |
| Malaysia | 19 (11 to 33) | 0.33 (0.18 to 0.56) | 25 (13 to 45) | 0.31 (0.16 to 0.55) | -0.13% (-0.2 to -0.07) |
| Maldives | 0 (0 to 0) | 0.25 (0.12 to 0.46) | 0 (0 to 0) | 0.26 (0.11 to 0.48) | 0.1% (0.08 to 0.12) |
| Mali | 25 (14 to 42) | 0.81 (0.45 to 1.35) | 61 (34 to 100) | 0.65 (0.36 to 1.07) | -0.6% (-0.66 to -0.55) |
| Malta | 0 (0 to 0) | 0.32 (0.19 to 0.51) | 0 (0 to 0) | 0.32 (0.18 to 0.54) | 0.21% (-0.2 to 0.62) |
| Marshall Islands | 0 (0 to 0) | 0.26 (0.12 to 0.48) | 0 (0 to 0) | 0.27 (0.12 to 0.5) | 0.14% (0.13 to 0.15) |
| Mauritania | 6 (3 to 10) | 0.75 (0.43 to 1.3) | 9 (5 to 16) | 0.57 (0.31 to 0.95) | -1% (-1.04 to -0.97) |
| Mauritius | 1 (0 to 2) | 0.26 (0.13 to 0.48) | 1 (0 to 1) | 0.27 (0.13 to 0.49) | 0.24% (0.1 to 0.38) |
| Mexico | 360 (275 to 491) | 1.14 (0.87 to 1.55) | 260 (184 to 385) | 0.76 (0.54 to 1.12) | -1.01% (-1.32 to -0.7) |
| Micronesia (Federated States of) | 0 (0 to 0) | 0.27 (0.12 to 0.49) | 0 (0 to 0) | 0.27 (0.12 to 0.5) | 0.02% (0.01 to 0.03) |
| Monaco | 0 (0 to 0) | 0.21 (0.09 to 0.38) | 0 (0 to 0) | 0.21 (0.09 to 0.38) | -0.05% (-0.08 to -0.02) |
| Mongolia | 9 (5 to 16) | 1.16 (0.6 to 2) | 10 (5 to 16) | 1.11 (0.59 to 1.92) | -0.12% (-0.25 to 0.02) |
| Montenegro | 2 (1 to 3) | 1.05 (0.53 to 1.82) | 1 (1 to 2) | 1.02 (0.52 to 1.74) | -0.12% (-0.15 to -0.09) |
| Morocco | 48 (25 to 82) | 0.54 (0.28 to 0.92) | 64 (29 to 113) | 0.65 (0.3 to 1.16) | 1.14% (0.74 to 1.55) |
| Mozambique | 50 (22 to 96) | 1.01 (0.45 to 1.95) | 126 (61 to 239) | 1.05 (0.51 to 2) | 0.55% (0.39 to 0.7) |
| Myanmar | 146 (43 to 310) | 1.03 (0.3 to 2.2) | 98 (38 to 183) | 0.61 (0.24 to 1.15) | -2.07% (-2.3 to -1.84) |
| Namibia | 3 (2 to 6) | 0.65 (0.36 to 1.13) | 5 (3 to 9) | 0.67 (0.35 to 1.16) | 0.05% (-0.04 to 0.14) |
| Nauru | 0 (0 to 0) | 0.27 (0.12 to 0.49) | 0 (0 to 0) | 0.28 (0.13 to 0.51) | 0.08% (0.06 to 0.1) |
| Nepal | 62 (29 to 108) | 0.9 (0.43 to 1.56) | 70 (37 to 120) | 0.73 (0.38 to 1.24) | -0.59% (-0.69 to -0.48) |
| Netherlands | 13 (8 to 19) | 0.4 (0.26 to 0.58) | 9 (5 to 14) | 0.29 (0.18 to 0.47) | -0.76% (-0.91 to -0.6) |
| New Zealand | 2 (1 to 4) | 0.26 (0.11 to 0.5) | 2 (1 to 4) | 0.19 (0.1 to 0.34) | -0.96% (-1.33 to -0.59) |
| Nicaragua | 8 (5 to 13) | 0.52 (0.31 to 0.83) | 8 (5 to 14) | 0.42 (0.23 to 0.72) | -0.63% (-0.69 to -0.56) |
| Niger | 26 (14 to 43) | 0.84 (0.45 to 1.4) | 57 (32 to 96) | 0.57 (0.31 to 0.96) | -1.3% (-1.41 to -1.19) |
| Nigeria | 257 (163 to 403) | 0.79 (0.5 to 1.25) | 626 (396 to 965) | 0.7 (0.44 to 1.08) | -0.37% (-0.42 to -0.33) |
| Niue | 0 (0 to 0) | 0.26 (0.12 to 0.5) | 0 (0 to 0) | 0.29 (0.14 to 0.53) | 0.18% (0.12 to 0.24) |
| North Macedonia | 6 (3 to 10) | 1.05 (0.54 to 1.84) | 4 (2 to 6) | 1.01 (0.51 to 1.76) | -0.14% (-0.15 to -0.13) |
| Northern Mariana Islands | 0 (0 to 0) | 0.25 (0.11 to 0.46) | 0 (0 to 0) | 0.26 (0.12 to 0.48) | 0.2% (0.15 to 0.24) |
| Norway | 5 (3 to 8) | 0.52 (0.28 to 0.88) | 5 (2 to 9) | 0.49 (0.25 to 0.88) | 0.56% (0.11 to 1.01) |
| Oman | 3 (1 to 5) | 0.46 (0.23 to 0.8) | 5 (2 to 8) | 0.47 (0.24 to 0.84) | 0.49% (0.34 to 0.64) |
| Pakistan | 453 (230 to 793) | 1.08 (0.56 to 1.89) | 920 (513 to 1489) | 1.16 (0.65 to 1.88) | 0.29% (0.19 to 0.4) |
| Palau | 0 (0 to 0) | 0.26 (0.12 to 0.48) | 0 (0 to 0) | 0.27 (0.12 to 0.5) | 0.09% (0.08 to 0.1) |
| Palestine | 4 (2 to 7) | 0.51 (0.26 to 0.88) | 8 (4 to 14) | 0.46 (0.24 to 0.81) | -0.22% (-0.33 to -0.11) |
| Panama | 4 (2 to 6) | 0.47 (0.29 to 0.75) | 5 (3 to 8) | 0.43 (0.24 to 0.72) | -0.19% (-0.26 to -0.12) |
| Papua New Guinea | 4 (2 to 7) | 0.27 (0.12 to 0.49) | 9 (4 to 17) | 0.28 (0.12 to 0.51) | 0.11% (0.1 to 0.12) |
| Paraguay | 5 (3 to 9) | 0.38 (0.21 to 0.64) | 12 (6 to 20) | 0.59 (0.31 to 0.95) | 1.65% (1.53 to 1.76) |
| Peru | 34 (18 to 59) | 0.44 (0.23 to 0.77) | 41 (21 to 72) | 0.44 (0.22 to 0.77) | -0.04% (-0.07 to -0.01) |
| Philippines | 412 (231 to 625) | 1.81 (1.02 to 2.75) | 479 (286 to 693) | 1.4 (0.84 to 2.04) | -0.43% (-0.67 to -0.2) |
| Poland | 134 (82 to 214) | 1.41 (0.86 to 2.25) | 46 (28 to 75) | 0.78 (0.48 to 1.28) | -2.1% (-2.33 to -1.86) |
| Portugal | 8 (5 to 12) | 0.3 (0.19 to 0.47) | 4 (2 to 7) | 0.24 (0.13 to 0.4) | -0.51% (-0.7 to -0.32) |
| Puerto Rico | 4 (2 to 7) | 0.37 (0.17 to 0.67) | 2 (1 to 4) | 0.38 (0.18 to 0.69) | 0.09% (0.06 to 0.11) |
| Qatar | 1 (0 to 1) | 0.57 (0.27 to 0.98) | 2 (1 to 3) | 0.45 (0.23 to 0.82) | -0.82% (-0.98 to -0.66) |
| Republic of Korea | 56 (30 to 97) | 0.42 (0.22 to 0.72) | 25 (12 to 45) | 0.36 (0.18 to 0.63) | -0.49% (-0.57 to -0.4) |
| Republic of Moldova | 13 (8 to 22) | 1.17 (0.7 to 1.92) | 6 (3 to 10) | 1.06 (0.61 to 1.76) | -0.32% (-0.4 to -0.24) |
| Romania | 62 (31 to 109) | 1.03 (0.52 to 1.82) | 33 (16 to 58) | 1.02 (0.5 to 1.82) | 0.02% (-0.03 to 0.06) |
| Russian Federation | 583 (390 to 884) | 1.73 (1.16 to 2.62) | 354 (211 to 578) | 1.37 (0.82 to 2.24) | -0.66% (-0.8 to -0.53) |
| Rwanda | 33 (13 to 72) | 1.25 (0.49 to 2.73) | 37 (19 to 69) | 0.78 (0.41 to 1.46) | -2.08% (-2.29 to -1.87) |
| Saint Kitts and Nevis | 0 (0 to 0) | 0.36 (0.17 to 0.66) | 0 (0 to 0) | 0.38 (0.18 to 0.69) | 0.13% (0.12 to 0.15) |
| Saint Lucia | 0 (0 to 0) | 0.38 (0.18 to 0.69) | 0 (0 to 0) | 0.42 (0.22 to 0.72) | 0.35% (0.29 to 0.41) |
| Saint Vincent and the Grenadines | 0 (0 to 0) | 0.4 (0.2 to 0.71) | 0 (0 to 0) | 0.39 (0.19 to 0.69) | 0.02% (-0.02 to 0.06) |
| Samoa | 0 (0 to 0) | 0.27 (0.12 to 0.49) | 0 (0 to 0) | 0.27 (0.12 to 0.5) | 0.04% (0.03 to 0.06) |
| San Marino | 0 (0 to 0) | 0.3 (0.17 to 0.5) | 0 (0 to 0) | 0.28 (0.14 to 0.48) | -0.11% (-0.19 to -0.04) |
| Sao Tome and Principe | 0 (0 to 1) | 0.81 (0.48 to 1.27) | 0 (0 to 1) | 0.55 (0.3 to 0.95) | -0.98% (-1.11 to -0.86) |
| Saudi Arabia | 23 (11 to 42) | 0.41 (0.2 to 0.74) | 34 (16 to 62) | 0.42 (0.2 to 0.78) | 0.14% (0.11 to 0.18) |
| Senegal | 22 (13 to 36) | 0.77 (0.44 to 1.25) | 37 (21 to 63) | 0.63 (0.36 to 1.08) | -0.57% (-0.66 to -0.49) |
| Serbia | 26 (14 to 45) | 1.14 (0.61 to 1.96) | 17 (8 to 30) | 1.03 (0.52 to 1.83) | -0.67% (-0.94 to -0.4) |
| Seychelles | 0 (0 to 0) | 0.3 (0.15 to 0.53) | 0 (0 to 0) | 0.26 (0.12 to 0.49) | 0.66% (0.3 to 1.02) |
| Sierra Leone | 11 (6 to 18) | 0.78 (0.43 to 1.29) | 20 (11 to 33) | 0.63 (0.36 to 1.02) | -0.49% (-0.56 to -0.41) |
| Singapore | 3 (2 to 5) | 0.4 (0.22 to 0.69) | 3 (2 to 5) | 0.42 (0.23 to 0.7) | 0.06% (-0.02 to 0.14) |
| Slovakia | 19 (11 to 32) | 1.38 (0.77 to 2.31) | 10 (5 to 18) | 1.2 (0.61 to 2.12) | -0.51% (-1.02 to 0) |
| Slovenia | 5 (3 to 8) | 1.15 (0.66 to 1.9) | 3 (1 to 5) | 0.97 (0.49 to 1.75) | -1.31% (-2.41 to -0.21) |
| Solomon Islands | 0 (0 to 1) | 0.27 (0.12 to 0.49) | 1 (0 to 1) | 0.28 (0.13 to 0.51) | 0.13% (0.12 to 0.14) |
| Somalia | 33 (14 to 76) | 1.13 (0.47 to 2.59) | 93 (40 to 198) | 1.12 (0.49 to 2.4) | -0.02% (-0.2 to 0.15) |
| South Africa | 90 (50 to 148) | 0.7 (0.39 to 1.15) | 105 (60 to 171) | 0.7 (0.4 to 1.13) | -0.16% (-0.33 to 0) |
| South Sudan | 23 (10 to 48) | 1.02 (0.43 to 2.14) | 46 (19 to 101) | 1.19 (0.48 to 2.59) | 0.37% (0.12 to 0.61) |
| Spain | 28 (17 to 44) | 0.29 (0.18 to 0.45) | 24 (13 to 41) | 0.33 (0.18 to 0.56) | 1.28% (0.86 to 1.71) |
| Sri Lanka | 19 (10 to 33) | 0.35 (0.18 to 0.6) | 17 (9 to 30) | 0.3 (0.16 to 0.53) | -0.41% (-0.51 to -0.3) |
| Sudan | 46 (23 to 80) | 0.61 (0.31 to 1.06) | 147 (60 to 257) | 0.93 (0.38 to 1.63) | 2.02% (1.66 to 2.39) |
| Suriname | 1 (0 to 1) | 0.4 (0.2 to 0.71) | 1 (0 to 1) | 0.42 (0.22 to 0.75) | 0.24% (0.19 to 0.29) |
| Sweden | 6 (3 to 9) | 0.34 (0.19 to 0.58) | 7 (3 to 13) | 0.38 (0.19 to 0.68) | 0.5% (0.36 to 0.65) |
| Switzerland | 4 (2 to 6) | 0.28 (0.16 to 0.46) | 3 (2 to 6) | 0.24 (0.12 to 0.43) | -1.02% (-1.25 to -0.78) |
| Syrian Arab Republic | 51 (25 to 95) | 0.97 (0.47 to 1.79) | 28 (14 to 49) | 0.6 (0.3 to 1.07) | -1.55% (-1.8 to -1.3) |
| Taiwan (Province of China) | 25 (16 to 37) | 0.43 (0.27 to 0.64) | 11 (7 to 17) | 0.33 (0.21 to 0.5) | -0.73% (-1.22 to -0.25) |
| Tajikistan | 37 (19 to 62) | 1.88 (0.97 to 3.21) | 53 (27 to 94) | 1.69 (0.86 to 3) | -0.77% (-0.99 to -0.55) |
| Thailand | 276 (137 to 523) | 1.52 (0.74 to 2.91) | 102 (59 to 178) | 0.87 (0.51 to 1.52) | -2.32% (-2.58 to -2.05) |
| Timor-Leste | 2 (1 to 3) | 0.65 (0.23 to 1.3) | 2 (1 to 4) | 0.48 (0.21 to 0.85) | -1.09% (-1.43 to -0.75) |
| Togo | 10 (6 to 16) | 0.71 (0.41 to 1.15) | 18 (11 to 30) | 0.62 (0.36 to 1) | -0.39% (-0.45 to -0.34) |
| Tokelau | 0 (0 to 0) | 0.26 (0.12 to 0.48) | 0 (0 to 0) | 0.28 (0.13 to 0.52) | 0.15% (0.1 to 0.2) |
| Tonga | 0 (0 to 0) | 0.26 (0.12 to 0.49) | 0 (0 to 0) | 0.27 (0.12 to 0.5) | 0.13% (0.12 to 0.14) |
| Trinidad and Tobago | 2 (1 to 3) | 0.48 (0.27 to 0.8) | 2 (1 to 3) | 0.53 (0.32 to 0.87) | 0.54% (0.4 to 0.69) |
| Tunisia | 15 (8 to 26) | 0.52 (0.28 to 0.9) | 15 (7 to 27) | 0.57 (0.28 to 1.01) | 0.63% (0.41 to 0.85) |
| Turkey | 156 (84 to 264) | 0.79 (0.43 to 1.34) | 101 (54 to 172) | 0.52 (0.28 to 0.89) | -1.17% (-1.27 to -1.06) |
| Turkmenistan | 16 (9 to 26) | 1.25 (0.67 to 2.04) | 18 (10 to 30) | 1.26 (0.7 to 2.12) | -0.01% (-0.06 to 0.05) |
| Tuvalu | 0 (0 to 0) | 0.27 (0.13 to 0.49) | 0 (0 to 0) | 0.27 (0.12 to 0.5) | 0.01% (-0.01 to 0.03) |
| Uganda | 50 (24 to 94) | 0.78 (0.38 to 1.45) | 143 (75 to 260) | 0.83 (0.43 to 1.51) | -0.1% (-0.28 to 0.07) |
| Ukraine | 163 (99 to 262) | 1.41 (0.86 to 2.27) | 81 (43 to 136) | 1.16 (0.63 to 1.97) | -0.78% (-0.89 to -0.66) |
| United Arab Emirates | 2 (1 to 4) | 0.53 (0.28 to 0.92) | 6 (3 to 10) | 0.47 (0.24 to 0.82) | -0.23% (-0.31 to -0.14) |
| United Kingdom | 61 (42 to 90) | 0.52 (0.36 to 0.77) | 52 (35 to 78) | 0.42 (0.29 to 0.63) | -0.11% (-0.33 to 0.11) |
| United Republic of Tanzania | 100 (45 to 194) | 1.02 (0.46 to 1.97) | 185 (96 to 348) | 0.85 (0.44 to 1.61) | -0.5% (-0.55 to -0.45) |
| United States of America | 296 (170 to 492) | 0.52 (0.3 to 0.87) | 316 (208 to 488) | 0.47 (0.31 to 0.73) | -0.95% (-1.45 to -0.45) |
| United States Virgin Islands | 0 (0 to 0) | 0.43 (0.22 to 0.74) | 0 (0 to 0) | 0.38 (0.18 to 0.69) | -0.17% (-0.29 to -0.05) |
| Uruguay | 4 (2 to 7) | 0.49 (0.25 to 0.87) | 4 (2 to 7) | 0.54 (0.3 to 0.92) | 0.18% (0.06 to 0.3) |
| Uzbekistan | 70 (34 to 125) | 0.99 (0.48 to 1.76) | 85 (42 to 149) | 1 (0.49 to 1.75) | 0.05% (0.03 to 0.06) |
| Vanuatu | 0 (0 to 0) | 0.26 (0.11 to 0.48) | 0 (0 to 1) | 0.27 (0.12 to 0.5) | 0.14% (0.13 to 0.15) |
| Venezuela (Bolivarian Republic of) | 48 (34 to 69) | 0.74 (0.51 to 1.06) | 63 (43 to 89) | 0.93 (0.65 to 1.32) | 1.08% (0.9 to 1.27) |
| Viet Nam | 78 (38 to 137) | 0.33 (0.16 to 0.58) | 93 (48 to 163) | 0.4 (0.21 to 0.71) | 1.1% (0.88 to 1.32) |
| Yemen | 28 (14 to 49) | 0.52 (0.27 to 0.92) | 88 (38 to 164) | 0.69 (0.3 to 1.28) | 1.18% (0.91 to 1.46) |
| Zambia | 31 (15 to 60) | 0.98 (0.47 to 1.92) | 68 (33 to 149) | 0.93 (0.45 to 2.04) | -0.47% (-0.66 to -0.29) |
| Zimbabwe | 24 (13 to 42) | 0.59 (0.32 to 1.01) | 43 (24 to 71) | 0.77 (0.43 to 1.25) | 1% (0.93 to 1.08) |

***Table S4. Predictions of incidence, mortality, and DALYs of pediatric stone disease in global stratified by sex, from 2022 to 2041.***

| year | ASIR_male | ASIR_female | ASMR_male | ASMR_female | ASDR_male | ASDR_female |
| --- | --- | --- | --- | --- | --- | --- |
| 2,022 | 184.47 (179.97 to 188.98) | 142.89 (139.01 to 146.78) | 0 (0 to 0) | 0 (0 to 0) | 0.81 (0.78 to 0.84) | 0.59 (0.55 to 0.62) |
| 2,023 | 184.38 (178.2 to 190.57) | 142.91 (137.62 to 148.21) | 0 (0 to 0) | 0 (0 to 0) | 0.79 (0.75 to 0.84) | 0.58 (0.53 to 0.62) |
| 2,024 | 184.27 (175.93 to 192.6) | 142.92 (135.83 to 150.02) | 0 (0 to 0) | 0 (0 to 0) | 0.78 (0.72 to 0.83) | 0.57 (0.51 to 0.62) |
| 2,025 | 184.14 (173.28 to 195) | 142.93 (133.72 to 152.14) | 0 (0 to 0) | 0 (0 to 0) | 0.76 (0.69 to 0.83) | 0.55 (0.48 to 0.62) |
| 2,026 | 184.03 (170.32 to 197.74) | 142.95 (131.33 to 154.56) | 0 (0 to 0) | 0 (0 to 0) | 0.74 (0.65 to 0.82) | 0.54 (0.45 to 0.62) |
| 2,027 | 183.92 (167.03 to 200.8) | 142.96 (128.67 to 157.25) | 0 (0 to 0) | 0 (0 to 0) | 0.72 (0.62 to 0.81) | 0.52 (0.42 to 0.62) |
| 2,028 | 183.77 (163.45 to 204.09) | 142.93 (125.74 to 160.13) | 0 (0 to 0) | 0 (0 to 0) | 0.69 (0.58 to 0.81) | 0.5 (0.39 to 0.62) |
| 2,029 | 183.55 (159.56 to 207.53) | 142.85 (122.54 to 163.16) | 0 (0 to 0) | 0 (0 to 0) | 0.67 (0.54 to 0.8) | 0.49 (0.36 to 0.62) |
| 2,030 | 183.26 (155.36 to 211.15) | 142.71 (119.09 to 166.34) | 0 (0 to 0) | 0 (0 to 0) | 0.65 (0.5 to 0.8) | 0.47 (0.32 to 0.62) |
| 2,031 | 182.93 (150.86 to 215) | 142.55 (115.37 to 169.73) | 0 (0 to 0) | 0 (0 to 0) | 0.63 (0.46 to 0.79) | 0.46 (0.29 to 0.62) |
| 2,032 | 182.6 (146.06 to 219.14) | 142.39 (111.39 to 173.4) | 0 (0 to 0) | 0 (0 to 0) | 0.61 (0.43 to 0.79) | 0.44 (0.26 to 0.62) |
| 2,033 | 182.27 (141 to 223.55) | 142.23 (107.15 to 177.32) | 0 (0 to 0.01) | 0 (0 to 0.01) | 0.59 (0.39 to 0.78) | 0.43 (0.23 to 0.62) |
| 2,034 | 181.95 (135.68 to 228.22) | 142.07 (102.68 to 181.47) | 0 (0 to 0.01) | 0 (0 to 0.01) | 0.57 (0.35 to 0.78) | 0.41 (0.2 to 0.63) |
| 2,035 | 181.62 (130.12 to 233.13) | 141.92 (97.99 to 185.84) | 0 (0 to 0.01) | 0 (0 to 0.01) | 0.55 (0.32 to 0.78) | 0.4 (0.17 to 0.63) |
| 2,036 | 181.3 (124.33 to 238.27) | 141.76 (93.09 to 190.42) | 0 (0 to 0.01) | 0 (0 to 0.01) | 0.53 (0.28 to 0.78) | 0.38 (0.14 to 0.63) |
| 2,037 | 180.98 (118.33 to 243.62) | 141.6 (87.99 to 195.21) | 0 (0 to 0.01) | 0 (0 to 0.01) | 0.51 (0.25 to 0.78) | 0.37 (0.11 to 0.63) |
| 2,038 | 180.65 (112.13 to 249.17) | 141.44 (82.7 to 200.17) | 0 (0 to 0.01) | 0 (0 to 0.01) | 0.5 (0.22 to 0.78) | 0.36 (0.08 to 0.64) |
| 2,039 | 180.33 (105.74 to 254.92) | 141.28 (77.24 to 205.32) | 0 (0 to 0.01) | 0 (0 to 0.01) | 0.48 (0.19 to 0.77) | 0.35 (0.06 to 0.64) |
| 2,040 | 180.01 (99.17 to 260.85) | 141.12 (71.6 to 210.64) | 0 (0 to 0.01) | 0 (0 to 0.01) | 0.46 (0.16 to 0.77) | 0.34 (0.03 to 0.64) |
| 2,041 | 179.69 (92.42 to 266.95) | 140.96 (65.8 to 216.13) | 0 (0 to 0.01) | 0 (0 to 0.01) | 0.45 (0.13 to 0.77) | 0.32 (0.01 to 0.64) |

***Table S5. Projected number of pediatric stone disease incidence, deaths and DALYs stratified by sex, 2022-2031.***

| year | Incidence_Num_male | Incidence_Num_female | mortality_Num_male | mortality_Num_female | DALYs_Num_male | DALYs_Num_female |
| --- | --- | --- | --- | --- | --- | --- |
| 2,022 | 1914477 (1508791 to 2320163) | 1390578 (990968 to 1790188) | 14 (0 to 1354231) | 4 (0 to 1573542) | 8359 (0 to 740081) | 5701 (0 to 854576) |
| 2,023 | 1924831 (1433523 to 2416138) | 1398322 (905580 to 1891063) | 9 (0 to 1898824) | 1 (0 to 2415790) | 8229 (0 to 1041878) | 5626 (0 to 1027409) |
| 2,024 | 1934641 (1197590 to 2671693) | 1406389 (671167 to 2160417) | 4 (0 to 2768825) | 0 (0 to 3511133) | 8039 (0 to 1736819) | 5507 (0 to 1546856) |
| 2,025 | 1943814 (913392 to 2974236) | 1414398 (465882 to 2481293) | 0 (0 to 3859074) | 0 (0 to 4721293) | 7785 (0 to 2557690) | 5341 (0 to 2198016) |
| 2,026 | 1952232 (673463 to 3232281) | 1422028 (365972 to 2753163) | 0 (0 to 5031252) | 0 (0 to 5918009) | 7496 (0 to 3265444) | 5147 (0 to 2778100) |
| 2,027 | 1959920 (593751 to 3415235) | 1428939 (287519 to 2940305) | 0 (0 to 6173463) | 0 (0 to 7014383) | 7205 (0 to 3780055) | 4947 (0 to 3213095) |
| 2,028 | 1964897 (532148 to 3538581) | 1433895 (221106 to 3062158) | 0 (0 to 7211015) | 0 (0 to 7962008) | 6921 (0 to 4133994) | 4750 (0 to 3518604) |
| 2,029 | 1967762 (477233 to 3623070) | 1437462 (162503 to 3142613) | 0 (0 to 8112153) | 0 (0 to 8750113) | 6648 (0 to 4376110) | 4563 (0 to 3728551) |
| 2,030 | 1968629 (429711 to 3679727) | 1439683 (112938 to 3193990) | 0 (0 to 8866948) | 0 (0 to 9382650) | 6388 (0 to 4532753) | 4388 (0 to 3863333) |
| 2,031 | 1967966 (393237 to 3713055) | 1440835 (76834 to 3220635) | 0 (0 to 9480128) | 0 (0 to 9873222) | 6148 (0 to 4613229) | 4230 (0 to 3933454) |
| 2,032 | 1967136 (371096 to 3726706) | 1441776 (57606 to 3225312) | 0 (0 to 9968000) | 0 (0 to 10242966) | 5935 (0 to 4626146) | 4090 (0 to 3948379) |
| 2,033 | 1963193 (358642 to 3722196) | 1440542 (51829 to 3210812) | 0 (0 to 10335463) | 0 (0 to 10498452) | 5736 (0 to 4583594) | 3961 (0 to 3918606) |
| 2,034 | 1957934 (353822 to 3706357) | 1438432 (55871 to 3183985) | 0 (0 to 10604811) | 0 (0 to 10663596) | 5552 (0 to 4503304) | 3844 (0 to 3857485) |
| 2,035 | 1951881 (353700 to 3684100) | 1435962 (66167 to 3150262) | 0 (0 to 10792725) | 0 (0 to 10755653) | 5381 (0 to 4398721) | 3737 (0 to 3775502) |
| 2,036 | 1945544 (355801 to 3659529) | 1433630 (79754 to 3114140) | 0 (0 to 10913992) | 0 (0 to 10789402) | 5222 (0 to 4280072) | 3642 (0 to 3680680) |
| 2,037 | 1938215 (357839 to 3633913) | 1431041 (94239 to 3077536) | 0 (0 to 10975314) | 0 (0 to 10771470) | 5070 (0 to 4152395) | 3553 (0 to 3577020) |
| 2,038 | 1935028 (363044 to 3617763) | 1431330 (108202 to 3048355) | 0 (0 to 11009944) | 0 (0 to 10729041) | 4935 (0 to 4029502) | 3480 (0 to 3474064) |
| 2,039 | 1931784 (368871 to 3605436) | 1431636 (120007 to 3022714) | 0 (0 to 11004141) | 0 (0 to 10649523) | 4807 (0 to 3905893) | 3413 (0 to 3368012) |
| 2,040 | 1928525 (369430 to 3598094) | 1432005 (128630 to 3001914) | 0 (0 to 10963701) | 0 (0 to 10538131) | 4685 (0 to 3783386) | 3352 (0 to 3260469) |
| 2,041 | 1925573 (364174 to 3597063) | 1432699 (133360 to 2987354) | 0 (0 to 10894617) | 0 (0 to 10400326) | 4569 (0 to 3663503) | 3298 (0 to 3152829) |
